# Glucocorticoid chronopharmacology promotes glucose metabolism in heart through a cardiomyocyte-autonomous transactivation program

**DOI:** 10.1172/jci.insight.182599

**Published:** 2024-11-22

**Authors:** Hima Bindu Durumutla, Ashok Daniel Prabakaran, Fadoua El Abdellaoui Soussi, Olukunle Akinborewa, Hannah Latimer, Kevin McFarland, Kevin Piczer, Cole Werbrich, Mukesh K. Jain, Saptarsi M. Haldar, Mattia Quattrocelli

**Affiliations:** 1 Molecular Cardiovascular Biology, Heart Institute, Cincinnati Children’s Hospital Medical Center and Department of Pediatrics, and; 2 Department of Pharmacology, Physiology and Neurobiology, University of Cincinnati College of Medicine, Cincinnati, Ohio, USA.; 3 Department of Cell Biology and Biochemistry, Brown University, Providence, Rhode Island, USA.; 4 Amgen Research, South San Francisco, California, USA.; 5 Gladstone Institutes, San Francisco, California, USA.; 6 Department of Medicine, Cardiology Division, UCSF, San Francisco, California, USA.

**Keywords:** Cardiology, Metabolism, Epigenetics, Glucose metabolism, Heart failure

## Abstract

Circadian time of intake gates the cardioprotective effects of glucocorticoid administration in both healthy and infarcted hearts. The cardiomyocyte-specific glucocorticoid receptor (GR) and its cofactor, Krüppel-like factor 15 (KLF15), play critical roles in maintaining normal heart function in the long term and serve as pleiotropic regulators of cardiac metabolism. Despite this understanding, the cardiomyocyte-autonomous metabolic targets influenced by the concerted epigenetic action of the GR/KLF15 axis remain undefined. Here, we demonstrated the critical roles of the cardiomyocyte-specific GR and KLF15 in orchestrating a circadian-dependent glucose oxidation program within the heart. Combining integrated transcriptomics and epigenomics with cardiomyocyte-specific inducible ablation of GR or KLF15, we identified their synergistic role in the activation of adiponectin receptor expression (*Adipor1*) and the mitochondrial pyruvate complex (*Mpc1/2*), thereby enhancing insulin-stimulated glucose uptake and pyruvate oxidation. Furthermore, in obese diabetic (*db/db*) mice exhibiting insulin resistance and impaired glucose oxidation, light-phase prednisone administration, as opposed to dark-phase prednisone dosing, restored cardiomyocyte glucose oxidation and improved diastolic function. These effects were blocked by combined in vivo knockdown of GR and KLF15 levels in *db/db* hearts. In summary, this study leveraged the circadian-dependent cardioprotective effects of glucocorticoids to identify cardiomyocyte-autonomous targets for the GR/KLF15 axis in glucose metabolism.

## Abstract 2

Depending on when it is taken during the day, the drug prednisone activates the heart to consume glucose to produce energy

## Graphical abstract

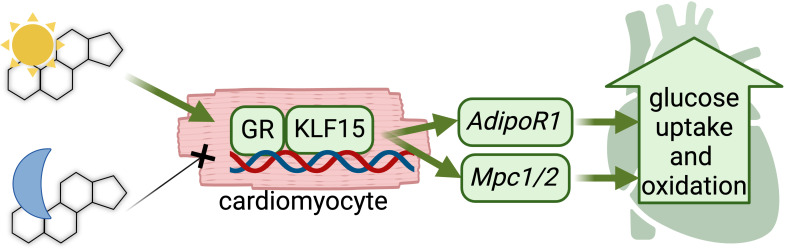

## Introduction

Chronic elevation of glucose levels in diabetes leads to pathological changes in mammalian cells, culminating in cellular and tissue damage, particularly affecting the cardiovascular system. Cardiovascular complications stand as the primary cause of morbidity and mortality among patients with diabetes mellitus (1). Moreover, individuals with metabolic disorders such as obesity and type 2 diabetes (T2D) are predisposed to diabetic cardiomyopathy, a condition of metabolic heart failure (2). T2D imposes metabolic stress on the heart, manifesting in — among others — 2 major defects: insulin resistance and impaired glucose oxidation. Insulin resistance in glucose uptake constitutes a critical metabolic insult in diabetic hearts and promotes a substrate imbalance that exacerbates diastolic dysfunction, i.e., loss of glucose oxidation with dominating lipid oxidation (3, 4). Moreover, the accumulation of ceramides in the diabetic myocardium further promotes a lipotoxic effect, compromising insulin sensitivity and glucose uptake (5). While previous studies have addressed the deficits in glucose oxidation and insulin sensitivity as single lines of action, comprehensive strategies to manage the metabolic abnormalities in diabetic cardiomyopathy remain elusive. Despite advancements in therapeutics approaches, including SGLT2 inhibitors and GLP1 agonists and/or lifestyle interventions, cardiovascular disease remains a leading cause of morbidity and mortality, accounting for approximately 40% of deaths in diabetic patients (6) — a statistic that continues to rise every year. Here we report a completely unanticipated role for the glucocorticoid receptor (GR) and its cofactor Krüppel-like factor 15 (KLF15) in regulating insulin-sensitive glucose uptake and oxidation in heart, addressing a critical clinical need to mitigate cardiovascular morbidity in these patients.

The GR is a potent metabolic regulator that acts as a transcription factor when activated by glucocorticoids. The cardiomyocyte-specific GR is important for heart function (7), but the cardiomyocyte-autonomous role of the GR in heart metabolism remains unknown. Notably, the glucocorticoid/GR axis is tightly linked to the circadian rhythm (8) and we previously reported that circadian time of intake gates the cardioprotective program enabled by the glucocorticoid in heart in a cardiomyocyte-autonomous, clock-dependent way (9). Among its targets, the GR activates its cofactor KLF15 (10), which regulates cardiac metabolism (11) and promotes circadian transcriptional programs in the heart (12). However, direct metabolic targets of the GR/KLF15 axis in cardiomyocytes are still remarkably unknown. Here we combine chronopharmacology with cardiomyocyte-specific inducible ablation and RNA-seq/ChIP-seq datasets to identify *Adipor1* and *Mpc1/2* as critical targets of GR and KLF15 in cardiac glucose utilization. Furthermore, we tested the relevance of these chronopharmacology targets for cardiac glucose oxidation and diastolic dysfunction improvements in obese diabetic mice. Our findings challenge the current paradigms in glucocorticoid biology and identify druggable mechanisms underlying the interplay between GR and KLF15. These insights may offer potential therapeutic strategies to combat metabolic inflexibility in heart failure.

## Results

### Light-phase dosing gates the glucocorticoid effects on cardiomyocyte glucose metabolism.

**Figure 1:**
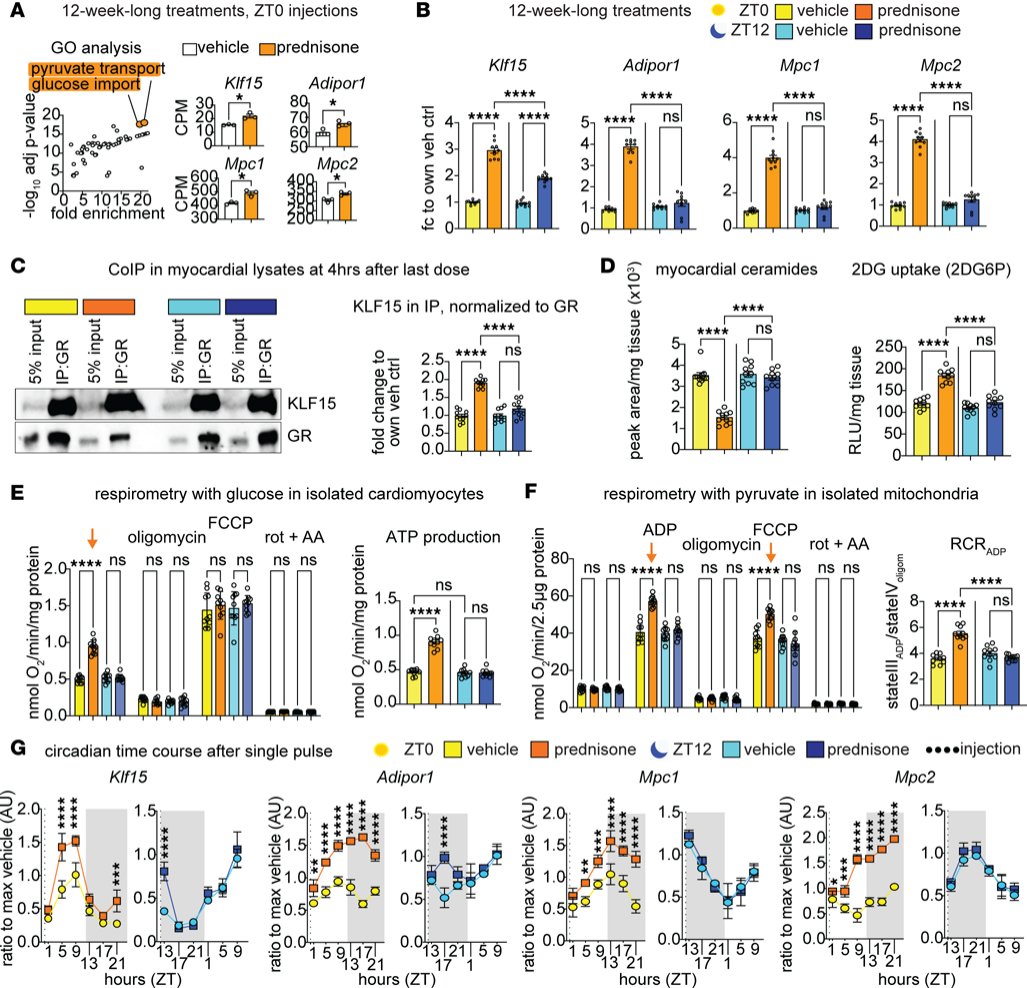
Light-phase glucocorticoid stimulation improves glucose uptake and oxidation in the heart. (**A**) Gene Ontology (GO) analysis revealed that ZT0 intermittent prednisone increased expression of *Klf15*, *Adipor1*, *Mpc1*, and *Mpc2* from the enriched pathways of pyruvate transport and glucose import in the WT hearts. (**B**) Upregulation of KLF15 by prednisone was higher after ZT0 than ZT12 dosing, and upregulation of *Adipor1*, *Mpc1*, and *Mpc2* was specific to ZT0 and blunted by ZT12 dosing. (**C**) Co-IPs in heart tissue showed that KLF15 interaction with GR was higher with ZT0 than ZT12 prednisone. (**D**) Consistent with *Adipor1* upregulation, ZT0 prednisone treatment decreased myocardial ceramide levels and increased insulin-dependent 2DG uptake in the heart. (**E**) After ZT0, but not ZT12, treatment, cardiomyocytes increased basal glucose-fueled respiration ex vivo (arrow) and ATP production. (**F**) Consistent with *Mpc1/2* upregulation, ZT0 treatment increased ADP-stimulated respiration (arrow) and respiratory control ratio with pyruvate in isolated mitochondria. (**G**) Circadian time-course qPCR analyses in myocardial tissues revealed oscillations of variable amplitude for *Klf15*, *Adipor1*, and *Mpc1/2* expression. The transactivation effect by a single prednisone pulse was pronounced and prolonged over the circadian cycle with ZT0 injections, while ZT12 prednisone effects were either transient or nonsignificant. Data are presented as mean ± SEM; histograms also show individual mouse values. *n* = 3 ♂/group in **A**, *n* = (5 ♀ + 5 ♂)/group in **B**–**G**. *P < 0.05; **P < 0.01; ***P < 0.001; ****P < 0.0001 by Welch’s *t* test (**A**) or 2-way ANOVA with Šidák’s post hoc test (**B**–**G**).

In our previous study, we reported that the cardioprotective circadian effects of the glucocorticoid prednisone in infarcted murine hearts are gated by the circadian time of intake (9). To elucidate the underlying molecular mechanisms, we analyzed RNA-seq datasets from uninjured (sham-operated) and myocardial tissues (GSE186875). Our analysis revealed that intermittent prednisone administration (1 mg/kg once per week intraperitoneally [i.p.] for 12 weeks) at zeitgeber time 0 (ZT0, lights on) significantly upregulated genes associated with increased glucose import and pyruvate transport pathways compared with vehicle controls. Among the genes involved in these pathways, we observed a remarkable upregulation of *Klf15*, *Adipor1*, and *Mpc1/2* following ZT0 prednisone stimulation (Figure 1A). KLF15 is a known circadian metabolic regulator of nutrient flux in the heart (12–15), yet its specific targets in conjunction with its cofactor GR (16) remain unknown. *Adipor1* encodes the adiponectin-activated ceramidase that is sufficient to promote insulin-sensitive glucose uptake (17, 18); *Mpc1* and -*2* encode the 2 subunits of the mitochondrial pyruvate carrier, essential for cardioprotective pyruvate oxidation (19–21). To test whether the steroid-driven transactivation of these genes was dependent on time of day, we treated wild-type (WT) C57BL/6J males with 12-week-long intermittent prednisone treatments at ZT0 versus ZT12, i.e., at start of light phase versus start of dark phase (Supplemental Figure 1A; supplemental material available online with this article; https://doi.org/10.1172/jci.insight.182599DS1). Consistent with our RNA-seq data, qPCR analysis showed that ZT0 prednisone significantly increased *Klf15*, *Adipor1*, *Mpc1*, and *Mcp2* compared with vehicle and, notably, the drug effect was significantly lower or blunted with ZT12 injections (Figure 1B). While we reported that circadian time of prednisone dosing did not change overall protein levels or nuclear translocation capacity of the GR in heart (9), we were intrigued by the time-of-dosing effects on *Klf15* expression. As KLF15 physically binds the GR as coactivator (16), we tested the extent to which the time-specific gating on *Klf15* upregulation converted to a parallel chronopharmacological effect on GR-KLF15 protein-protein interaction. At 4 hours after the last drug dose, we found that indeed KLF15 binding to GR was increased by ZT0 prednisone to a higher extent than that induced by ZT12 prednisone (Figure 1C). To assess the functional implications of *Adipor1*, *Mpc1*, and *Mcp2* upregulation in cardiac metabolic remodeling, we quantitated myocardial ceramide abundance through mass spectrometry (MS), glucose uptake through 2-deoxyglucose (2DG) assay in vivo, glucose oxidation through glucose-fueled respirometry in isolated cardiomyocytes, and pyruvate-fueled respiratory control ratio (RCR) (22) in myocardium-derived isolated mitochondria, analyzing data as aggregated (Figure 1, C–F) and disaggregated by sex (Supplemental Figure 1, B–E). In line with *Adipor1* upregulation, ZT0, but not ZT12, prednisone decreased myocardial ceramides (Figure 1C). With ZT0 prednisone administration, we observed a significant increase in insulin-dependent 2DG uptake (Figure 1D), glucose-fueled respiration, and calculated ATP production in cardiomyocytes (Figure 1E). Consistent with the upregulation of *Mpc1/2*, ZT0 treatment also elevated ADP-stimulated respiration in mitochondria, increasing pyruvate-fueled RCR (Figure 1F). Ceramide clearance and the effects in cardiomyocytes and mitochondria were sex independent (Supplemental Figure 1, B–E). Regarding the gene expression trends, we sought to gain insight into expression oscillations and time-of-intake effects in heart. We performed a circadian time-course qPCR analysis in myocardial tissues after a single prednisone pulse at ZT0 versus ZT12, to compare circadian trends in the absence of chronic secondary effects. Expression curves revealed oscillations of variable amplitude for *Klf15*, *Adipor1*, and Mpc1/2 in the WT myocardium, with the most pronounced circadian mRNA acrophases for *Klf15* in the light-phase and *Mpc1* during the light-dark phase transition (Figure 1G). The transactivation effect by a single prednisone pulse was pronounced and prolonged over the circadian cycle with ZT0 injections, while ZT12 prednisone effects were either transient or nonsignificant (Figure 1G). Considering the upregulation of *Klf15* appeared central in the circadian-specific prednisone-GR program, we asked whether the cardiomyocyte clock was mediating the time-specific effect. Indeed, we previously showed that the cardiomyocyte-specific brain and muscle Arnt-like protein 1 (BMAL1, gene symbol *Arntl*) was required for the ZT0 prednisone-specific pro-energetic effects in heart (9). Following the ChIP-seq indications of GR-BMAL1 interplay previously garnered in muscle (23), we looked for GR elements (GREs; ACAnnnTGT) and BMAL1 E-Box sites (CACGTG) in the *Klf15* proximal promoter within 300–900 bp from each other and found a proximal promoter region with a GRE and E-Box at approximately 600 bp from each other (Supplemental Figure 1F). Through ChIP-qPCR in myocardial tissue at 4 hours after the last drug injection, we found that GR occupancy on the GRE was increased by treatment regardless of time of intake, while BMAL1 occupancy on the E-Box was increased specifically by ZT0 and not ZT12 prednisone (Supplemental Figure 1F). We leveraged the mice with inducible cardiomyocyte-specific *Arntl* knockout (BMAL1-KO) we previously reported (9) to assess the extent to which BMAL1 was required for the ZT0 prednisone effect on cardiac *Klf15* transactivation. After tamoxifen and washout following prior conditions (9), BMAL1-KO versus BMAL1-WT (i.e., not ablated) littermates were treated with 12-week-long ZT0 prednisone or vehicle treatments. In the heart, cardiomyocyte BMAL1 ablation blocked the ZT0 prednisone effects on GR occupancy of the *Klf15* promoter GRE and on *Klf15* mRNA upregulation. The data were further validated by parallel analyses in skeletal muscle, where BMAL1 was not ablated and ZT0 prednisone effects were indeed not blocked (Supplemental Figure 1G). Taken together, our findings indicate that circadian time of intake gates the prednisone effects on *Klf15, Adipor1*, and *Mpc1/2* oscillations, as well as ceramide clearance, insulin-sensitive glucose uptake, and pyruvate oxidation in the uninjured heart.

### Generation of ChIP-seq and RNA-seq datasets for the cardiomyocyte-autonomous GR and KLF15.

**Figure 2:**
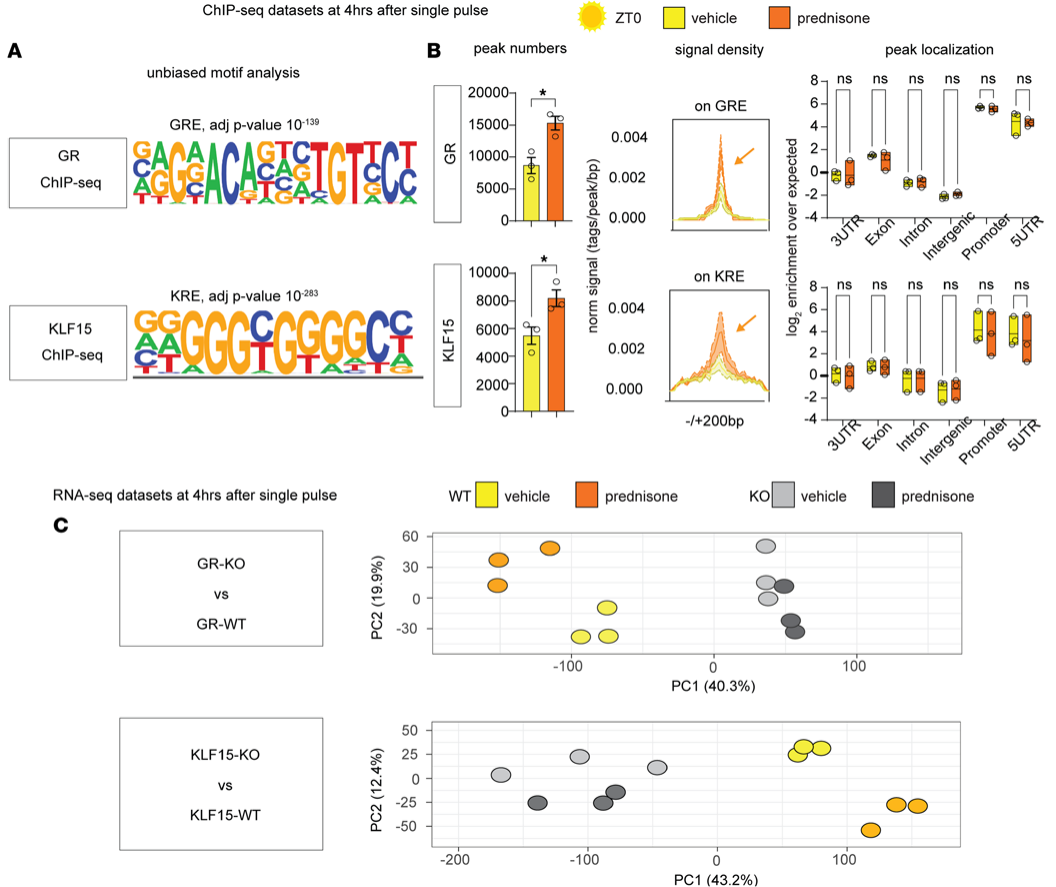
ChIP-seq and RNA-seq datasets reveal nonredundant mediator effect of both cardiomyocyte-specific GR and KLF15 on ZT0 glucocorticoid gene programs in the heart. (**A**) Validation of ChIP-seq datasets through unbiased motif analysis. (**B**) Prednisone increased peak numbers and canonical epigenomic peak signal (arrows) for both GR and KLF15 without changes in relative enrichment in genomic localization, which was enriched in promoter and 5′ UTRs for both factors. (**C**) PCA analyses show sample clustering according to drug and genotype variables. *n* = 3 ♂/group. In **B** and **C**, Welch’s *t* test was used to assess peak number and 2-way ANOVA with Šidák’s post hoc test was used to evaluate peak localization. *P < 0.05.

Given the expected upregulation of *Klf15* and activation of GR by prednisone treatment, we tested the role of the GR/KLF15 axis in mediating the upregulation of *Adipor1* and *Mpc1/*2 by light-phase glucocorticoids specifically in cardiomyocytes. For this, we sought to garner unbiased evidence of GR and KLF15 epigenomic occupancy and transcriptional effects in heart. To profile epigenomic occupancy of GR and KLF15 in myocardial tissue, we conducted parallel ChIP-seq experiments 4 hours after a single pulse of ZT0 prednisone administration, adapting the method reported for muscle in our previous study (23). We focused on epigenomic shifts after a single drug pulse to avoid secondary effects of chronic drug administration. Notably, due to the lack of ChIP-seq–grade commercial antibodies for KLF15, we performed the immunoprecipitations in myocardial tissue of *Klf15*^3xFLAG/wt^-knockin mice, which were previously reported (24). Our unbiased motif analysis validated the specificity of GR and KLF15 immunoprecipitations, revealing high enrichment for each factor’s canonical binding motifs (GREs and KLF response elements, KREs; Figure 2A). The epigenomic activity of both GR and KLF15 was activated by ZT0 prednisone in myocardium, as indicated by increased peak number and signal intensity over canonical sites genome wide (Figure 2B). However, there were no discernible drug-induced changes in peak localization, which remained highly enriched in promoter transcription start sites (TSSs) (Figure 2B).

Next, to gain proof of requirement for target gene expression trends, we collected RNA-seq datasets from hearts of cardiomyocyte-specific inducible KO versus WT mice for each factor, using the same tamoxifen and washout protocol (Supplemental Figure 2A). For GR-KO we used the mice we previously reported (9), comparing hearts from *Myh6-MerCreMer^+/–^;Nr3c1^fl/fl^* (GR-KO) versus *Myh6-MerCreMer^+/–^;Nr3c1^wt/wt^* (GR-WT) mice. Similarly, for KLF15-KO mice we compared *Myh6-MerCreMer^+/–^;Klf15^fl/fl^* (KLF15-KO) versus *Myh6-MerCreMer^+/-^;Klf15^wt/wt^* (KLF15-WT) (Supplemental Figure 2B), using the previously reported *Klf15*-floxed allele (12). As for the ChIP-seq datasets, the RNA-seq datasets were garnered from hearts collected 4 hours after a single ZT0 prednisone pulse. Principal component analysis (PCA) of the RNA-seq datasets clustered samples based on drug treatment and cardiomyocyte genotype for either *GR* or *Klf15* (Figure 2C), suggesting a nonredundant requirement for both factors in the transcriptional program enabled by ZT0 prednisone in heart. We therefore generated datasets to unbiasedly probe the extent to which the cardiomyocyte-specific GR/KLF15 axis regulates *Adipor1* and *Mpc1/2* expression in the heart.

### Cardiomyocyte GR and KLF15 are independently required for the ZT0 prednisone effect on Adipor1 and Mpc1/2 transactivation, insulin-driven glucose uptake, and pyruvate oxidation.

**Figure 3:**
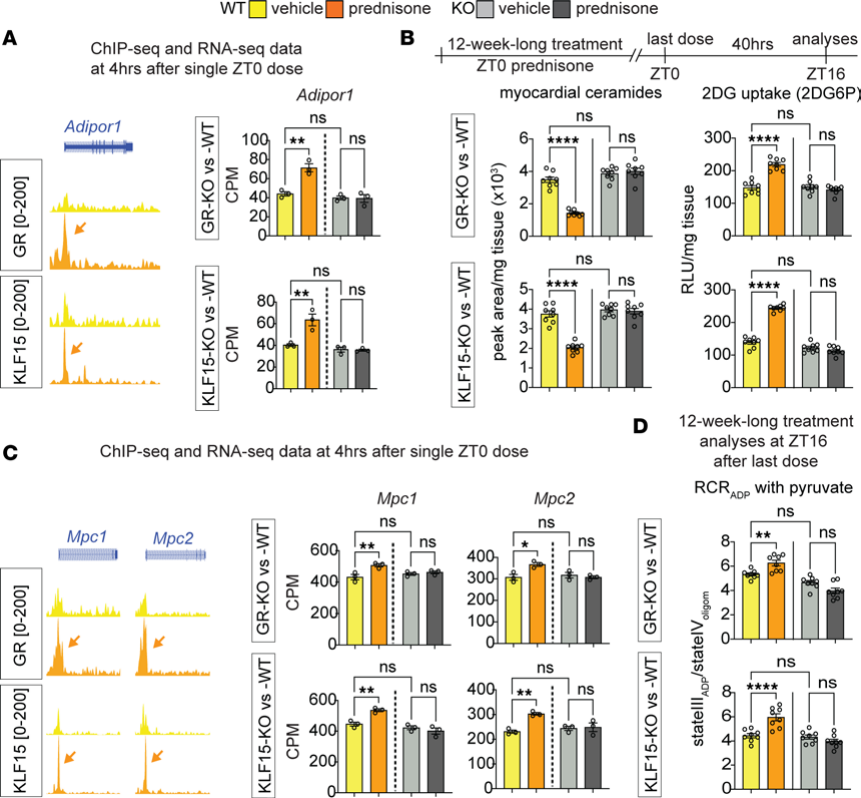
Cardiomyocyte GR and KLF15 are required for the ZT0 prednisone effects on gene transactivation, insulin sensitization, and pyruvate oxidation in heart. (**A**) GR and KLF15 showed steroid-sensitive peaks on the *Adipor1* TSS (arrows). Drug-induced upregulation was blunted by inducible cardiomyocyte-specific ablation of either GR or KLF15. (**B**) Transcriptional/metabolic effects of treatment appeared durable beyond the resting phase, as GR and KLF15 were both required for the treatment effects on myocardial ceramide content reduction and insulin-stimulated glucose uptake increment in the heart in the active phase (ZT16). (**C** and **D**) Similarly, GR and KLF15 were required for the drug effect on *Mpc1/2* transactivation, and for the chronic treatment effects on pyruvate oxidation in the heart in the active phase. Data are presented as mean ± SEM; histograms also show individual mouse values. *n* = 3 ♂/group in **A** and **C**; *n* = 8 ♂/group in **B** and **D**. *P < 0.05; **P < 0.01; ****P < 0.0001 by 2-way ANOVA with Šidák’s post hoc test.

We first tested the extent to which *Adipor1* was a direct transactivation target of GR and KLF15 in the heart downstream of ZT0 prednisone. ChIP-seq peak tracks revealed steroid-sensitive peaks on the *Adipor1* proximal promoter for both GR and KLF15. RNA-seq data showed that the inducible cardiomyocyte-specific ablation of either GR or KLF15 blocked the drug-induced upregulation of *Adipor1*, indicating a nonredundant requirement for both factors in a cardiomyocyte-autonomous fashion (Figure 3A). Based on the motifs unveiled by the unbiased motif analysis of ChIP-seq datasets (ACAnnnTGT for GRE, GGGCGGGG for KRE), we found GRE and KRE motifs in the proximal promoter of *Adipor1* and validated the ChIP-seq and RNA-seq indications through ChIP-qPCRs and qPCRs (Supplemental Figure 2C). In accordance with the trends in *Adipor1* transactivation, cardiomyocyte-specific inducible ablation of either GR or KLF15 blocked the chronic ZT0 prednisone effects on ceramide reduction and 2DG uptake in the heart. The transcriptional and metabolic effects of treatment appeared durable and extended to not only the light phase, but also the active phase of treated mice (Figure 3B and Supplemental Figure 2C). Similarly, we found steroid-sensitive peaks on the proximal promoters of *Mpc1* and *Mpc2*, with ZT0 prednisone transactivation of both genes blocked by cardiomyocyte-specific ablation of either GR or KLF15 (Figure 3C). We found GRE and KRE motifs in the 5′ UTRs of both *Mpc1* and *Mpc2* and validated GR-KLF15 occupancy and gene upregulation through ChIP-qPCRs and qPCRs (Supplemental Figure 2C). In accordance with the trends in *Mpc1/2* transactivation, the chronic treatment–driven gain in pyruvate oxidation was also blocked by the inducible gene ablation and the KLF15-dependent chronic treatment effects were applicable to both active and resting phases of mice (Figure 3D and Supplemental Figure 2D). Taken together, these findings demonstrate that the ZT0-specific prednisone stimulation of glucose uptake and oxidation in the heart is mediated by the cardiomyocyte-autonomous epigenetic activity of both GR and KLF15.

**Figure 4:**
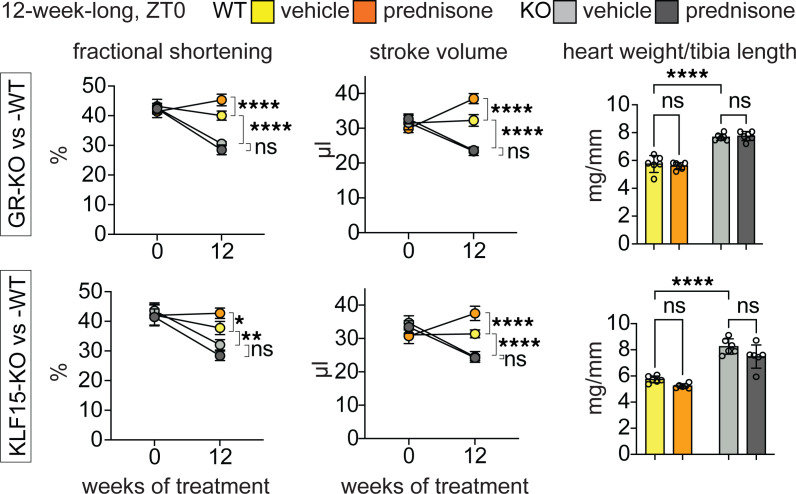
Functional cardiac assessments for chronic ZT0 prednisone effects on uninjured hearts after cardiomyocyte gene ablation. Either GR-KO or KLF15-KO induced significant losses in fractional shortening, stroke volume, and gains in heart weight/tibia length compared with WT. In WT uninjured hearts, treatment increased stroke volume and fractional shortening, with no significant trends in heart weight. As expected, treatment had no sizable effects in KO hearts. Data are presented as mean ± SEM; histograms also show individual mouse values. *n* = 6 ♂/group. *P < 0.05; **P < 0.01; ****P < 0.0001 by 3-way ANOVA with Šidák’s post hoc test (curves) or 2-way ANOVA with Šidák’s post hoc test (histograms).

We then assessed heart function through echocardiography in our cohorts at the start and end of 12-week-long ZT0 prednisone treatments. We primarily focused on fractional shortening, stroke volume, and heart weight/tibia length, as those parameters were previously shown to be impacted by both GR and KLF15 constitutive (postnatal, not inducible) ablations in heart (25, 26). In our own mice after inducible ablation and in the absence of prednisone, KO of either factor significantly depressed fractional shortening and stroke volume, while increasing heart mass, when compared with WT controls. In WT uninjured hearts, ZT0 prednisone treatment increased stroke volume and fractional shortening, with no significant trends in normalized heart weight. As expected, treatment had no sizable effects in KO hearts (Figure 4). In line with the trends in fractional shortening and stroke volume, KO of either factor enlarged the systolic left ventricle diameter (LVID) and induced nonsignificant declines in ejection fraction and gains in diastolic left ventricle diameter (LVIDd). In WT hearts, treatment induced a small significant reduction in LVIDs, a nonsignificant trend in increased ejection fraction, and no appreciable changes in LVIDd. In KO hearts, treatment had no sizable effects. Heart rate during the echocardiographic measurements, i.e., under anesthesia, was not changed by either KO or treatment (Supplemental Figure 3). Therefore, inducible ablation of either GR or KLF15 after normal growth impacted function in the uninjured heart, blocking the treatment-driven gains in stroke volume.

### Light-phase glucocorticoid intermittence rescues glucose oxidation and diastolic dysfunction in diabetic hearts.

**Figure 5:**
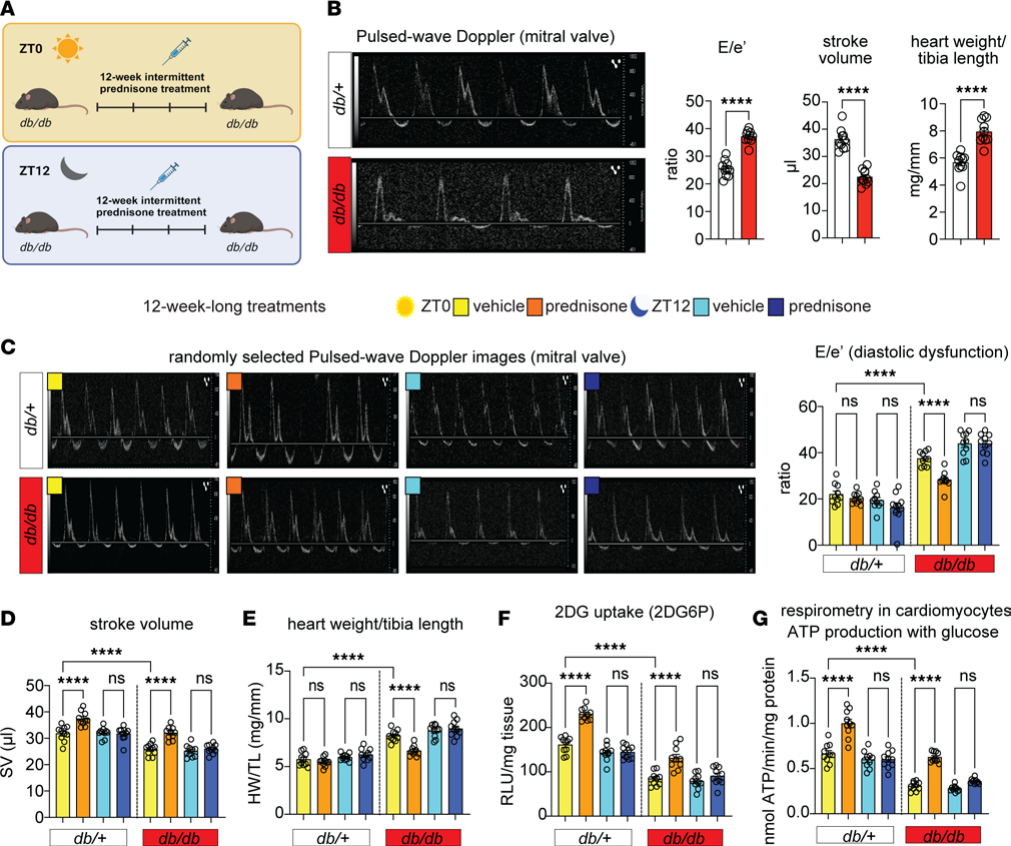
ZT0 glucocorticoid treatment rescues glucose oxidation and diastolic function in *db/db* mice. (**A**) Schematic showing the 12-week-long prednisone treatment of *db/db* and their littermate controls (*db*/+) at ZT0 and ZT12. (**B**) Representative images of pulse wave Doppler echocardiography for mitral valve flow, and quantitation of baseline defects in E/e′, stroke volume, and heart mass in *db/db* mice. (**C**) ZT0 treatment decreased E/e′ in *db/db* hearts to *db/+*-like levels, while ZT12 treatment had no sizable effects. (**D**) ZT0, but not ZT12, treatment increased stroke volume in both *db/db* and *db/+* mice. (**E**) Cardiac hypertrophy was alleviated in *db/db* hearts by ZT0, but not ZT12 treatment. (**F**) Treatment increased glucose uptake in myocardial tissue, measured as 2DG uptake, in both *db/+* and *db/db* hearts only with ZT0 dosing. (**G**) Treatment increased glucose-fueled ATP production calculated from Seahorse curves in isolated cardiomyocytes in both *db/+* and *db/db* mice according to time of intake (ZT0 but not ZT12), increasing *db/db* values to control-like levels. Data are presented as mean ± SEM; histograms also show individual mouse values. *n* = (5 ♀ + 5 ♂)/group. ****P < 0.0001 by Welch’s *t* test (**B**) or 3-way ANOVA with Šidák’s post hoc test (**C**–**G**).

**Figure 6:**
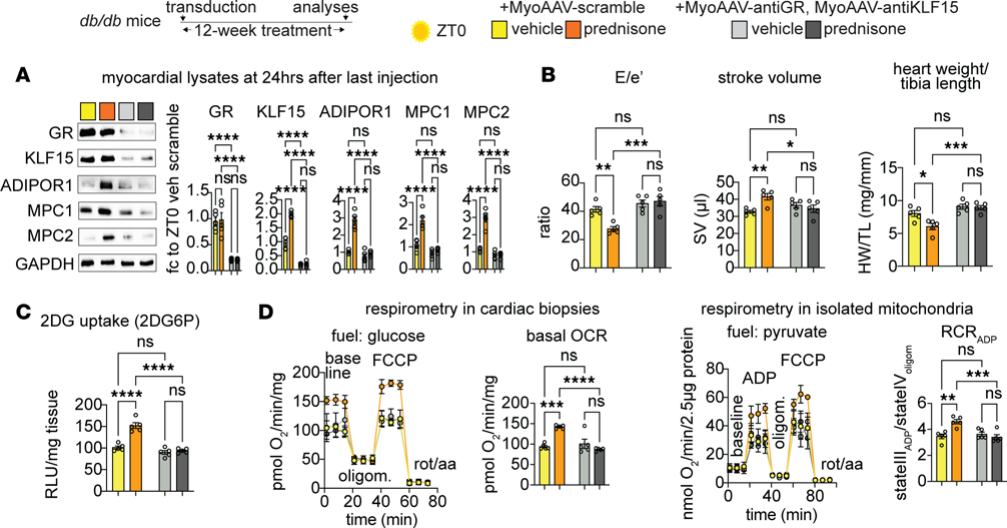
Knockdown of GR and KLF15 in *db/db* hearts in vivo blocks the ZT0 prednisone effects. (**A**) Representative Western blot showing knockdown of GR and KLF15 at 12 weeks after transduction and ZT0 prednisone regimen start, as well as blunting of the treatment effect on ADIPOR1, MPC1, and MPC2 protein level upregulation in hearts of *db/db* mice transduced with the knockdown vectors compared with mice transduced with scramble vectors. (**B** and **C**) Knockdown MyoAAV combination blocked the treatment effects on diastolic dysfunction (E/e′), stroke volume, cardiac hypertrophy (heart weight/tibia length), and insulin-driven glucose uptake (2DG6P). (**D**) Treatment increased glucose-fueled respiration (oxygen consumption rate, OCR) in permeabilized cardiac biopsies and pyruvate-fueled respiration (respiratory control ratio, RCR) in isolated myocardial mitochondria in scramble-, but not knockdown-transduced, hearts. Data are presented as mean ± SEM; histograms also show individual mouse values. *n* = 5 ♂/group. *P < 0.05; **P < 0.01; ***P < 0.001; ****P < 0.0001 by 2-way ANOVA with Šidák’s post hoc test.

We hypothesized that combining the ZT0 prednisone effects on insulin sensitization and pyruvate oxidation could serve as a “two-hit” approach to enhance a functional glucose oxidation program in the diabetic heart. Given that insulin resistance and impaired glucose oxidation are key metabolic dysfunctions in T2D diabetic cardiomyopathy (3, 4), we aimed to evaluate the relevance of this ZT0 prednisone program in a diabetic cardiomyopathy model. To address this, we employed 6-month-old *db/db* obese diabetic mice (Figure 5A) (27, 28) and their *db/+* littermates as nonobese, nondiabetic parallel control cohort. At baseline, we reconfirmed previously reported defects in diastolic function (mitral valve flow impairment and increased E/e′; ref. 28), systolic function (decreased stroke volume; refs. 29, 30), and cardiac hypertrophy (increased heart mass to tibia length ratio; ref. 31) in *db/db* mice versus *db/+* controls (Figure 5B). Importantly, these parameters were rescuable in *db/db* mice through experimental interventions reactivating glucose uptake and oxidation (29–32). After treatment with intermittent prednisone at ZT0 versus ZT12 for 12 weeks, ZT0 prednisone alleviated diastolic dysfunction, stroke volume impairment, and cardiac hypertrophy in *db/db* mice toward control *db/+* levels, while the treatment effects were blocked by ZT12 injections (Figure 5, C–E). Only with ZT0 dosing did treatment increase glucose uptake in myocardial tissue, measured as 2DG uptake, in both *db/+* and *db/db* hearts (Figure 5F). Also, compared with isochronic vehicle, ZT0, but not ZT12, prednisone treatment increased glucose-fueled basal respiration and ATP production in *db/db* cardiomyocytes toward control-like *db/+* levels (Figure 5G). Notably, none of these parameters showed significant sexual dimorphism in their effects (Supplemental Figure 4A). Treatments had no sizable effects on body weight beyond the expected genotype-dependent increase (Supplemental Figure 4B). Furthermore, we checked blood pressure through tail cuff measurements on the day after the last injection, considering the hypertensive phenotype of *db/db* mice (33) and the hypertensive potential of chronic glucocorticoid treatments (34). We conducted the tail cuff measurements in the active phase (ZT16) to avoid potential confounding dips induced by sleep or rest. None of the treatments had sizable effects on blood pressure or heart rate during blood pressure measurements beyond the expected *db/db*-related mild hypertension in both systole and diastole (Supplemental Figure 4C). In light of the ZT0-specific effects of prednisone on KLF15-GR interaction in WT nondiabetic hearts, we tested the extent to which the chronic ZT0 versus ZT12 regimens changed GR or KLF15 nuclear translocation in *db/db* hearts. We performed Western blotting (WB) for GR and KLF15 in *db/db* myocardial lysate fractions at 30 minutes after the last injection of ZT0 versus ZT12 prednisone at the end of the 12-week-long treatment. We found that time of intake did not sizably change the rate of GR nuclear translocation, while it did impact KLF15 translocation, which was specifically increased by ZT0 prednisone (Supplemental Figure 5A). Indeed, in line with the model of GR-KLF15 transactivation of *AdipoR1* and *Mpc1/2*, we found that at 24 hours after the last drug injection those proteins were increased by ZT0 prednisone — but not ZT12 prednisone — compared with vehicle in *db/db* hearts (Supplemental Figure 5B). We also found analogous trends in the glucose transporters GLUT1 and GLUT4 (Supplemental Figure 5B), which were convergent with the glucose uptake reactivation in those hearts and consistent with prior seminal findings with *Klf15* overexpression (35). Finally, we sought to validate that the ZT0 prednisone effects in *db/db* hearts were dependent on GR and KLF15. To that goal, we used MyoAAV-serotyped vectors containing either a scrambled or anti-*Nr3c1* (MyoAAV-antiGR) or anti-*Klf15* (MyoAAV-antiKLF15) shRNA under the U6 promoter. The MyoAAV serotype maximizes tropism of transduction for adeno-associated virus (AAV) vectors in vivo to striated muscles (36). To maximize knockdown, 3 different vectors each with a different shRNA were combined per gene knockdown. At 6 months of age, male *db/db* mice were injected retro-orbitally (r.o.) with 6 × 10^12^ vg/mouse MyoAAV-scramble or 1 × 10^12^ vg/mouse/vector MyoAAV-antiGR (3 vectors) plus MyoAAV-antiKLF15 (3 vectors) immediately before starting the 12-week-long ZT0 prednisone versus vehicle intermittent regimens. At the endpoint, WB in myocardial tissue confirmed GR and KLF15 knockdown (~80% protein reduction) and showed that the knockdown vectors blocked or blunted the ZT0 prednisone effects on upregulation of ADIPOR1 and MPC1/2 protein levels in *db/db* hearts (Figure 6A). Accordingly, the combinatorial GR+KLF15 knockdown blocked the treatment effects on diastolic dysfunction (E/e′), stroke volume, cardiac hypertrophy (heart weight/tibia length), and insulin-driven glucose uptake (Figure 6, B and C). Moreover, treatment increased glucose-fueled respiration (oxygen consumption rate, OCR) in permeabilized cardiac biopsies and pyruvate-fueled respiration (RCR) in isolated myocardial mitochondria in scramble- but not knockdown-transduced hearts (Figure 6D). Taken together, these data indicate the potential for prednisone chronopharmacology to reactivate glucose oxidation and alleviate dysfunction in obese diabetic hearts by engaging the GR-KLF15 interplay in the heart in vivo.

## Discussion

The GR plays a pivotal role in metabolic regulation, acting as a transcription factor when activated by glucocorticoids. While the importance of a cardiomyocyte-specific GR in heart function is recognized (7), its autonomous role in heart metabolism remains unclear. Our previous work has elucidated 2 critical time dimensions influencing the outcomes of GR activation: (i) circadian time of intake and (ii) chronic frequency of intake. Light-phase dosing of prednisone in mice has shown benefits in heart function compared with dark-phase dosing (9). Intermittent once-weekly dosing reversed the dysmetabolic effects of chronic daily dosing and improved exercise tolerance (37). In obese diabetic mice, endogenous glucocorticoids are elevated in response to metabolic stress and yet they maintain circadian oscillations, albeit dimmed (38). This will be an important point to elucidate for the translation of our findings into pharmacological strategies for diabetic cardiomyopathy, although pilot clinical data with prednisone chronopharmacology in patients with muscular dystrophies showed promising improvements in lean mass and exercise tolerance (39).

Despite the known regulation of GR activity by the circadian clock (40, 41), the specific targets of glucocorticoid chronopharmacology for functional cardiac metabolic remodeling remain poorly elucidated. Our findings from this study reveal one such mechanism: the cardiomyocyte-autonomous requirement for the GR/KLF15 axis in facilitating light-phase-specific glucocorticoid transactivation of *Adipor1* and the MPC complex genes in the heart. ADIPOR1, an adiponectin-responsive ceramidase (42), is generally linked to pro-metabolic effects and plays a crucial role in insulin-sensitive glucose uptake by degrading ceramides (18, 43). Ceramides accumulate in the heart with obesity and/or T2D, promoting cardiac insulin resistance and the related cardiac dysfunction (44). It is important to note that overt, unbalanced reactivation of glucose uptake in the insulin-resistant heart causes unwanted side effects, as shown by cardiomyocyte GLUT4 overexpression in diabetic hearts (45). From that perspective, our findings with the simultaneous upregulation of both ADIPOR1 and the MPC complex could offer a model to reactivate both glucose uptake and downstream oxidation in a balanced fashion.

MPC expression is downregulated in failing human hearts (46) and transcriptional MPC abundance directly promotes cardioprotective oxidation of pyruvate and its precursor glucose in the myocardium (19, 20, 46). Accordingly, MPC activity is decreased in the diabetic heart too (47). It was remarkable to observe a strong concerted epigenetic program by the cardiomyocyte-specific GR and KLF15 on both subunit genes for the MPC, which indeed resulted in pyruvate oxidation trends in line with increased glucose oxidation.

The variable of circadian time of drug intake inevitably questions the time gating on not only basic mechanisms, but also chronic effects with respect to the circadian cycle. Here, the ZT0-prednisone-gated interaction between GR and KLF15 stimulated cardiomyocyte-autonomous transcriptional/metabolic effects that extended to both resting and active phases of mice after chronic treatment. In other words, a repetitive series of circadian-specific drug stimulations supported a virtuous program that improved glucose handling in cardiomyocytes throughout the circadian cycle. This is an emerging aspect in various contexts of chronopharmacology, including the heterogeneous landscape of chrono-immunology for cardiovascular health (48). Particularly in the context of diabetic cardiomyopathy, these findings open the question for future studies of which chronological regimens can be complemented to concertedly leverage the endogenous rhythms of all the critical cell compartments of the heart, including cardiomyocytes, fibroblasts, endothelial cells, and immune cells.

### Limitations of the study.

While sufficiently powered for overall trends with chronological treatments and gene ablation, our cohort numbers were limited and likely underpowered for omics analyses and sex-specific differences. The limited numbers used for RNA-seq and ChIP-seq were mitigated by dataset overlay and further validation through 2 different cardiomyocyte-specific inducible KO models. The limited numbers of mice in our sex-disaggregated analyses probably limited the detection of sexual dimorphism, which will have to be addressed for the pathways in play here with appropriate cohort numbers and hormonal manipulations in future studies. Despite the mechanistic evidence offered by cardiomyocyte-specific GR and KLF15 inducible ablation in nondiabetic conditions and by the in vivo knockdown in *db/db* hearts, in this study we did not directly address the requirement for the effects on insulin sensitivity and glucose oxidation per se in the metabolic remodeling of the obese diabetic heart. We believe this is beyond the scope of this investigation.

### Conclusions and overall impact.

In summary, our study challenges the current paradigm of glucocorticoid cardiotoxicity by identifying a specific mechanism that mediates the circadian-gated glucocorticoid benefit on cardiomyocyte glucose oxidation, with functional implications for diabetic cardiomyopathy.

## Methods

### Sex as biological variable.

We performed the bulk of our wild-type and diabetic mice experiments in both males and females, analyzing and reporting the physiological, molecular and histological assessments as sex-aggregated in main figures and sex-disaggregated in the supplemental figures. We did not find sizable sex-specific patterns. Therefore, we performed our unbiased omics-based screenings and subsequent KO- or knockdown-based proofs of requirement only in male mice to minimize variability and overall mouse number used.

### Animal handling and treatments.

Mice were housed in a pathogen-free facility in accordance with the American Veterinary Medical Association (AVMA). Consistent with the ethical approvals, all efforts were made to minimize suffering. Euthanasia was performed through carbon dioxide inhalation followed by cervical dislocation and heart removal.

Mice were maintained on a 12-hour light/dark cycle, and diet/pharmacological treatments were initiated at approximately 12 weeks of age. The following mice were obtained and interbred from The Jackson Laboratory: WT C57BL/6J mice (strain 000664), *Myh6-MerCreMer* mice (strain 005657), *GR*-flox mice (strain 021021), *Arntl*-flox (BMAL1-flox; strain 007668), and *db/db* (strain 000642). *Klf15*-flox mice (strain 029787) and *Klf15*^3xFLAG/wt^ mice (24) were provided in-house. Gene ablation was induced with i.p. tamoxifen at approximately 8 weeks of age (20 mg/kg/day for 5 days; T5648, Sigma-Aldrich) and analyses were performed after 4 weeks of tamoxifen washout, i.e., at approximately 16 weeks of age.

Weekly prednisone treatment consisted of once-weekly i.p. injection of prednisone (1 mg/kg; P6254, Sigma-Aldrich). The injectable solution was diluted from a stock (5 mg/mL) in dimethyl sulfoxide (DMSO; D2650, Sigma-Aldrich) in a 50-μL volume. Injections were conducted either at the beginning of the light phase (ZT0; lights on) or at the beginning of the dark phase (ZT12; lights off). Tissues were harvested 4 hours after injection for epigenetic/transcriptional assays and 24 hours after injection for metabolic/physiologic assays. All in vivo, ex vivo, and postmortem analyses were conducted blinded to treatment group.

For systemic AAV experiments, 6-month-old *db/db* mice were administered either vehicle or the 12-week-long prednisone regimen at the beginning of the light phase (ZT0; lights on). These mice were further injected retro-orbitally with either 6 × 10^12^ genome copies/injection MyoAAV-scramble shRNA or 1 × 10^12^ genome copies/injection for each of the MyoAAV-*Nr3c1*shRNA and MyoAAV-*Klf15*shRNA vectors (Vector Builder cargo vectors VB010000-0023jze, VB240517-1300eyt, VB240517-1305cxu, VB240517-1306ywz, VB900144-1448eer, VB240517-1307ced, and VB240517-1308zdv; anti-*Nr3c1* shRNA sequences: AGGATTGCAAGCCTCTTATTT, TGAGATTCGAATGACTTATAT, TTTGCTCCTGATCTGATTATT; anti-*Klf15* shRNA sequences: CATTTCTGCTTCCCTGAATTT, CTACCCTGGAGGAGATTGAAG, ACCGAAATGCTCAGTGGGTTA; U6 promoter for all scramble and shRNA constructs) while under inhaled isoflurane anesthesia. All MyoAAV injections were diluted in sterile PBS. To prepare and isolate AAV virions, we followed the procedures we previously reported (49).

### RNA-seq and qPCR analyses.

RNA-seq was conducted on RNA extracted from left ventricle myocardial tissue. Each heart was immediately snap frozen in 1 mL TRIsure (BIO-38033, Bioline) using liquid nitrogen. RNAs from each heart were repurified using an RNeasy Mini Kit (74104, QIAGEN). RNA-seq was performed by the DNA Core at Cincinnati Children’s Hospital Medical Center (CCHMC). Total RNA (150–300 ng) determined by Qubit (Q33238, Invitrogen) high-sensitivity spectrofluorometric measurement was poly-A selected and reverse transcribed using Illumina’s TruSeq stranded mRNA library preparation kit (20020595, Illumina). Each sample was fitted with one of 96 adapters containing a different 8-base molecular barcode for high-level multiplexing. After 15 cycles of PCR amplification, completed libraries were sequenced on an Illumina NovaSeq 6000, generating 20 million or more high quality 100-base long paired-end reads per sample. A quality control check on the fastq files was performed using FastQC (https://github.com/s-andrews/FastQC). Upon passing basic quality metrics, the reads were trimmed to remove adapters and low-quality reads using default parameters in Trimmomatic v0.33 (https://github.com/timflutre/trimmomatic). In the next step, transcript/gene abundance was determined using kallisto (v0.43.1). The trimmed reads were then mapped to the mm10 reference genome using default parameters with strandness (R for single-end and RF for paired-end) option in Hisat2 v2.0.5 (https://daehwankimlab.github.io/hisat2/). In the next step, transcript/gene abundance was determined using kallisto v0.43.1 (https://github.com/pachterlab/kallisto). We first created a transcriptome index in kallisto using Ensembl cDNA sequences for the reference genome. This index was then used to quantify transcript abundance in raw counts and counts per million (CPM). Differential expression (FDR < 0.05) was quantitated through DESeq2 (https://bioconductor.org/packages/release/bioc/html/DESeq2.html). PCA was conducted using ClustVis (https://biit.cs.ut.ee/clustvis/). Gene ontology pathway enrichment was conducted using the Gene Ontology analysis tool (https://geneontology.org/).

Total RNA was extracted from cryo-pulverized liver tissue and human induced pluripotent stem cell–derived hepatocyte-like cells using Trizol (Thermo Fisher Scientific) and 1 μg RNA was reverse transcribed using SuperScript IV VILO Master Mix (11756050, Thermo Fisher Scientific). RT-qPCRs were conducted in 3 replicates using 1× SYBR Green Fast qPCR machine (Bio-Rad; thermal profile: 95°C for 15 seconds; 60°C for 30 seconds, 40 times; melting curve). The 2^–ΔΔCt^ method was used to calculate relative gene expression. GAPDH was used as the internal control. Primers were selected among validated primer sets from the MGH PrimerBank (https://pga.mgh.harvard.edu/primerbank/) as follows (gene, forward, reverse): *Klf15,* GAGACCTTCTCGTCACCGAAA*,* GCTGGAGACATCGCTGTCAT*; Adipor1,* TCTTCGGGATGTTCTTCCTGG*,* TTTGGAAAAAGTCCGAGAGACC*; Mpc1,* CTCCAGAGATTATCAGTGGGCG*,* GAGCTACTTCGTTTGTTACATGGC*; Mpc2,* CTCAGTCCACTGTGTTGATGGC*,* ATCCGAAACAGCTGAGAGGCTC.

### MS analysis of ceramides in myocardial tissue.

Untargeted lipidomics was performed on myocardial tissue from mice treated for 12-week-long intermittent treatments with vehicle or weekly prednisone. Whole hearts were snap-frozen and then ground to powder with a dry ice–chilled mortar and pestle. Frozen samples were sent on dry ice to the Mass Spectrometry Core at CCHMC, where LC-MS and data analysis were performed. Briefly 10 μL of sample was added to 30 μL of internal standard, except for the blank calibrator. Then, 300 μL of butanol/methanol (3:1) was added to samples and vortexed for 10 minutes. Next, 150 μL of heptane/ethyl acetate (3:1) was added to the sample and vortexed for 5 minutes. The contents of the sample were then transferred to a large glass tube, to which 150 μL of heptane/ethyl acetate was added and vortexed for 5 minutes. Then, 300 μL of 1% acetic acid was added to the original tube to wash, vortexed for 5 minutes, and then added back to the glass tube. The glass tube was further vortexed for 5 minutes and rested for 5 minutes. The upper organic phase of approximately 200 μL was transferred to a new glass tube. To this, 320 μL heptane/ethyl acetate (3:1) was added to the water phase, vortexed for 5 minutes, and allowed to sit for 5 minutes. Next, 280 μL of upper organic phase (second extract) was transferred to the first extract. This step was repeated once. The upper organic phase (third extract) of 100 μL was combined with the other extracts and dried down with nitrogen in a heating block set to 40°C. The resuspended lipid extracts (2 μL) were loaded and separated through a Xevo QTOF G2-S mass spectrometer (Waters Corp). All calculations were performed using QuanLynx (https://www.waters.com/nextgen/en/library/application-notes/2006/quanlynx-application-manager.html).

### ChIP-seq.

Whole hearts were cryopowdered using a liquid nitrogen–cooled RETSCH Cryomill. The cryopowdered tissue was then fixed in 10 mL of 1% paraformaldehyde (PFA) for 30 minutes at room temperature with gentle nutation. Fixation was quenched with 1 mL of 1.375 M glycine (BP381-5, Thermo Fisher Scientific) with gentle nutation for 5 minutes at room temperature. After centrifugation at 3,000*g* for 5 minutes at 4°C, the pellet was resuspended in cell lysis buffer as per reported conditions, supplementing the cell lysis buffer with cytochalasin B (3 μg/mL) and rotating for 10 minutes at 4°C. Nuclei were pelleted at 300*g* for 10 minutes at 4°C and subsequently processed following the reported protocol with the adjustment of adding cytochalasin B (3 μg/mL) into all solutions for chromatin preparation and sonication, antibody incubation, and wash steps. Chromatin was then sonicated for 15 cycles (30 seconds, high power, 30-second pause, and 500 μL volume) in a water bath sonicator set at 4°C (Bioruptor 300, Diagenode). After centrifuging at 10,000*g* for 10 minutes at 4°C, sheared chromatin was checked by agarose gel for a shear band of between approximately 150 and approximately 600 bp. Two micrograms of chromatin were kept for pooled input controls, whereas approximately 50 μg of chromatin was used for each pull-down reaction in a final volume of 2 mL rotating at 4°C overnight. Primary antibodies were as follows: rabbit polyclonal anti-GR (A2164, ABclonal) and mouse monoclonal anti-FLAG M2 (F1804, Sigma-Aldrich). Chromatin complexes were precipitated with 100 μL of Dynabeads M-280 (sheep anti-mouse, 11202D; sheep anti-rabbit, 11204; Thermo Fisher Scientific). After washes and elution, samples were treated with proteinase K (19131, QIAGEN) at 55°C, and cross-linking was reversed through overnight incubation at 65°C. DNA was purified using a MinElute purification kit (28004, QIAGEN) and quantitated using Qubit reader and reagents. Library preparation and sequencing were conducted at the CCHMC DNA Core, using TruSeq ChIP-seq library prep (with size exclusion) on approximately 10 ng of chromatin per ChIP sample or pooled inputs and HiSeq 50-bp was conducted using HOMER software v4.10 (http://homer.ucsd.edu/homer/) after aligning fastq files to the mm10 mouse genome using bowtie2 (https://bowtie-bio.sourceforge.net/bowtie2/index.shtml). PCA was conducted using ClustVis. Heatmaps of peak density were imaged with TreeView3 (https://github.com/ckeil0689/treeview3). Peak tracks were imaged through WashU epigenome browser (https://epigenomegateway.wustl.edu/browser). Gene ontology pathway enrichment was conducted using the gene ontology analysis tool.

For ChIP-qPCR analyses, muscle chromatin was immunoprecipitated following the conditions for ChIP-seq. Input and IP chromatins were diluted 100-fold and assayed using the qPCR conditions. The regions identified in the *Klf15* promoter were a GRE at –1167 bp from the TSS (primers, Fw TCTAGACAGCTGGGGCATCT, Rev GACAGACCTTCCTTCCTGGC) and a BMAL1 E-Box at –560 bp from the TSS (primers, Fw AAGCACAGACTCCTTCCGTG, Rev CGCTACCCTAGACTTCTGCG). Additional regions and primers: *Adipor1* GRE, Fw AGCTGGGGTTCTAGGACACT, Rev GCTGCTCCCCCTTTAAGTGT; *Adipor1* KRE, Fw AAGATTGCCTTCCCAGCTCC, Rev GGAGGGGCCGGAAATGTTTA; *Mpc1* GRE, Fw GCTCTTGGTTAAGGCGACCT, Rev ACACATGATTGTCCGGTCCC; *Mpc1* KRE, Fw GCTCTTGGTTAAGGCGACCT, Rev ACACATGATTGTCCGGTCCC; *Mpc2* KRE, Fw ATAGCGAGATCCAAGCCAGC, Rev CTAGGGATCGACAGCAGCAG; *Mpc2* GRE, Fw CTGCTGCTGTCGATCCCTAG, Rev GCTGAGACCAGACAGACACC. Signal in IP chromatin was quantitated as percentage of input signal per qPCR calculations.

### 2DG-6-phosphate assay.

For measuring the uptake of 2-DG-6-phosphate, a Glucose Uptake-Glo assay (J1341, Promega) was utilized to inject 1 mM 2DG into mice for 30 minutes before euthanasia. After 30 minutes, the whole heart tissues were harvested and stored at –80°C until use. Next, the reagents from the kit were combined for reaction mixtures according to manufacturer’s instructions and incubated at room temperature for 1 hour. The frozen heart tissues were crushed into a fine powder and 20–50 mg of tissue powder was used for the assay. After a 1-hour incubation of the reaction mixture, 25 μL of neutralization buffer was added to reaction mixture. Next, 125 μL of reaction mixture and neutralization buffer was added to powdered heart tissue and incubated for 1 hour. After incubation, the samples were centrifuged for 5 minutes at 10,000*g* and 125 μL of the supernatant was loaded onto a 96-well plate for luminescence readings.

### Respirometry on cardiac biopsies, cardiomyocytes, and isolated mitochondria.

For cardiac biopsies, whole hearts were harvested and multiple transverse cuts were performed with a scalpel in the left ventricular region with small scissors. Size-matched tissue biopsies were prepared as thin as possible (~0.5 mm^2^) to cover the bottom of the Seahorse plate’s well. The biopsies were permeabilized in 100 μg/mL saponin solution (SAE0073, Sigma-Aldrich) and incubated in XF base medium with 10 mM glucose, 2 mM L-glutamine, and 1 mM pyruvate (G7021, G7513, P2256, Sigma-Aldrich) at 37°C in a non-CO_2_ incubator for 30 minutes. The standard Seahorse protocol was followed with basal reads and injections of oligomycin (495455-10 MG, Millipore), FCCP, and rotenone (557368-1 GM, Millipore)/antimycin A (A674-50 MG, Sigma-Aldrich) following reported conditions. Basal OCR was calculated as the baseline value (average of 3 consecutive reads) minus the value after rotenone/antimycin addition (average of 3 consecutive reads). Basal OCR values were normalized to total protein content using a Bradford assay.

For cardiomyocytes, respirometry was performed on freshly isolated cells. Cardiomyocytes were isolated using using the following procedures. In anesthetized mice, the descending aorta was cut, and the heart was immediately flushed by injecting 7 mL prewarmed EDTA buffer (130 mM NaCl, 5 mM KCl, 0.5 mM NaH_2_PO_4_, 10 mM HEPES, 10 mM glucose, 10 mM 2,3-butanedione monoxime [BDM], 10 mM taurine, 5 mM EDTA, pH 7.8) into the right ventricle. Immediately afterwards, the ascending aorta was clamped using Reynolds forceps, and the heart was removed and kept in the 60-mm dish with prewarmed EDTA buffer. The digestion process was started by injecting 10 mL prewarmed EDTA buffer into the apex of the left ventricle. The heart was then transferred to the 60-mm dish with perfusion buffer (130 mM NaCl, 5 mM KCl, 0.5 mM NaH_2_PO_4_, 10 mM HEPES, 10 mM glucose, 10 mM BDM, 10 mM taurine, 1 mM MgCl_2_, pH 7.8) and 3 mL perfusion buffer was injected to flush out the EDTA buffer. Then, the heart was transferred to another 60-mm dish with prewarmed collagenase buffer (contains 180 U/mL collagenase II) and 30–40 mL collagenase buffer was injected into the apex of the left ventricle. Then, the heart was cut from the clamp and the ventricular myocardial tissue was pulled gently into small pieces using forceps and further dissociated by gentle pipetting. The cardiomyocyte suspension was passed through a 200-μm mesh, and the digestion was stopped by adding 5 mL of stop buffer (perfusion buffer supplemented with 5% sterile FBS). Cardiomyocytes were then centrifuged at 100*g* for 3 minutes, resuspended in the stop buffer with increasing concentrations of calcium (100 μM, 400 μM, and 1000 μM), re-centrifuged, and then plated in laminin-coated Seahorse plates in culture media (contains [per 250 mL]: 2.45 g Hanks’ salt, 5 mL nonessential amino acids, 2.5 mL MEM Vitamin Solution, 0.0875 g NaHCO_3_, 2.5 mL 10× PenStrep, 1 mg/mL BSA). After 1-hour incubation, the culture media were aspirated and Seahorse media were added for respirometry, which started after an additional 1-hour equilibrium in a CO_2_-free incubator. The standard Seahorse protocol was followed with basal reads and injection of oligomycin, FCCP, and rotenone/antimycin following reported conditions. Basal OCR was calculated as baseline value (average of 3 consecutive reads) minus the value after rotenone/antimycin addition (average of 3 consecutive reads). Basal OCR values were normalized to total protein content, assayed through homogenization in each well after the Seahorse run and Bradford assay.

Mitochondrial ATP production rate was calculated as OCR_ATP_ × 2 (mol O in mol O_2_) × 2.75 (Seahorse P/O), where OCR_ATP_ is the difference between baseline and oligomycin OCR. Nutrients: 10 mM glucose or 10 mM pyruvate; inhibitors: 0.5 μM rotenone plus 0.5 μM antimycin A. RCR values were obtained from isolated mitochondria from myocardial tissue. Tissues from the left ventricle were harvested and cut up into very fine pieces. The minced tissue was placed in a 15 mL conical tube (188261, USA Scientific) and 5 mL of MS-EGTA buffer with 1 mg trypsin (T1426-50 MG, Sigma-Aldrich) was added to the tube. MS-EGTA buffer consisted of 225 mM mannitol (M0214-45, ChemProducts), 75 mM sucrose (100892, Millipore), 5 mM HEPES (15630–080, Gibco), and 1 mM EGTA (E14100–50.0, RPI), pH 7.4 when ice-cold. The tube was quickly vortexed, and the tissue was left submerged in the solution. After 2 minutes, 5 mL of MS-EGTA buffer with 0.2% BSA (A-421-250, Goldbio) was added to the tube to stop the trypsin reaction. The tube was inverted several times to mix and then set to rest. Once the tissue had settled to the bottom of the tube, 3 mL of buffer was aspirated and the remaining solution and tissue was transferred to a 10 mL glass tissue homogenizer (89026–382, Avantor). Once sufficiently homogenized, the solution was transferred back into the 15 mL conical tube and spun at 1,000*g* for 5 minutes at 4°C. After spinning, the supernatant was transferred to a new 15 mL conical tube. The supernatant in the new tube was then centrifuged at 12,000*g* for 10 minutes at 4°C to pellet the mitochondria. The supernatant was discarded from the pellet and the pellet was then resuspended in 7 mL of MS-EGTA buffer and centrifuged again at 12,000*g* for 10 minutes at 4°C. After spinning, the supernatant was discarded, and the mitochondria were resuspended in 1 mL of Seahorse medium (103335–100, Agilent) supplemented with 5 mM pyruvate (P2256-100G, Sigma-Aldrich). After protein quantitation using a Bradford assay (5000001, Bio-Rad), 2.5 μg of mitochondria were dispensed per well in 180 μL total volume and allowed to equilibrate for 1 hour at 37°C. ADP (20 μL of 5 mM; 01905, Sigma-Aldrich), 50 μM oligomycin, 100 μM carbonyl cyanide-*p*-trifluoromethoxyphenylhydrazone (C3463, TCI), and 5 μM rotenone/antimycin A were added to drug ports A, B, and C, respectively. At baseline and after each drug injection, samples were read 3 consecutive times. RCR was calculated as the ratio between state III (OCR after ADP addition) and uncoupled state IV (OCR after oligomycin addition). All Seahorse measurements were conducted blinded to treatment groups.

### Western blotting.

Protein analyses were performed on approximately 50 μg of total lysate from the whole heart. Cyropulverized cardiac tissue was incubated in RIPA buffer (89900, Thermo Fisher Scientific) supplemented with 1× protease/phosphatase inhibitor (78440, Thermo Fisher Scientific) for 30 minutes and sonicated for 10 seconds twice. The samples were then centrifuged at 10,000*g* for 10 minutes at 4°C. Supernatant containing the protein was transferred into a new tube and used as the total lysate. For total cell lysates from culture cells, cells were harvested and resuspended in RIPA buffer containing 1× protease and phosphatase inhibitors. Lysates were incubated for 30 minutes and centrifuged at 10,000*g* for 10 minutes at 4°C. The supernatant was used as the total cell lysate. The protein concentrations of the supernatants were determined using the Pierce BCA Protein Assay kit (23227, Thermo Fisher Scientific). Equal amounts of protein were separated using SDS-PAGE and transferred to a PVDF membrane (1620174, Bio-Rad). Membranes were blocked in 5% milk in TBST for 1 hour at room temperature and then incubated overnight at 4°C with the following primary antibodies: mouse anti-KLF15 monoclonal antibody (sc-271675, Santa Cruz Biotechnology), rabbit anti-GAPDH monoclonal antibody (A19056, ABclonal), rabbit anti–histone H3 polyclonal antibody (A2348, ABclonal), mouse anti-GR monoclonal antibody (sc-393232, Santa Cruz Biotechnology), rabbit anti-AdipoR1 polyclonal antibody (A16527, ABclonal), rabbit anti-Mpc1 polyclonal antibody (A20195, ABclonal), rabbit anti-Mpc2 polyclonal antibody (A20196, ABclonal), rabbit anti-GLUT1 polyclonal antibody (A11208, ABclonal), or rabbit anti-GLUT4 polyclonal antibody (A7637, ABclonal). Primary antibodies were followed by incubation with HRP-conjugated secondary Peroxidase AffiniPure Goat Anti-Rabbit IgG (H+L) (111-035-003, Jackson ImmunoResearch Labs) for 1 hour at room temperature. Immunoreactive bands were visualized by chemiluminescence using Pierce Enhanced Chemiluminescent Western blotting substrate (32106, Thermo Fisher Scientific).

### Nuclear and cytoplasmic fraction analysis.

The separation of nuclear and cytoplasmic protein analysis was performed using the NE-PER nuclear and cytoplasmic extraction kit (78835, Invitrogen). Briefly, 100 mg of the heart tissues was homogenized, and 1 mL of CERI solution was added and vortexed vigorously on high setting for 15 seconds. Following incubation on ice for 10 minutes, 55 μL of ice-cold CERII solution was added, vortexed, and incubated for 1 minute and centrifuged at 16,000*g* for 5 minutes. The supernatant containing the cytoplasmic fraction was separated and collected into a new 1.5 mL Eppendorf tube. The insoluble material was suspended in 500 μL of ice-cold NER solution. The sample was then placed on ice for 40 minutes and vortexed every 10 minutes for 1 second. Finally, the samples were centrifuged at 16,000*g* for 10 minutes. The supernatant containing the nuclear fraction was transferred into a new 1.5 mL Eppendorf tube. All samples were stored at –80°C until use. The protein concentration was determined by Bio-Rad protein assay as described above. Once the protein concentration was determined, Western blotting analysis was carried out as described above.

### Measurement of blood pressure by tail-cuff plethysmography.

The CODA 8 system (Kent Scientific) was used to noninvasively measure blood pressure in mice through tail-cuff recordings. It was factory calibrated, and standard settings and procedures were adhered to as recommended by the manufacturer. Before beginning the experiments, the occlusion and VPR cuffs were routinely checked. Experiments were carried out in a controlled environment maintained at 22°C ± 2°C, where mice were acclimated for 1 hour prior to measurements. Mice were gently guided into the restraint tubes and tube end holders were adjusted at the tail’s base, with the VPR sensor cuff positioned next to it. Heating pads provided with the CODA 8 system were preheated to 33°C–35°C. Mice were warmed on these pads for 5 minutes before and during the blood pressure recording. To obtain blood pressure readings, the occlusion cuff was inflated to 25 mmHg and then deflated over 20 seconds. The VPR sensor cuff detected the changes in tail volume as blood flow returned deflation, with a minimal volume change set at 15 μL. Each recording session included 15–25 cycles of inflation and deflation, with the first 5 cycles used for acclimation and excluded from the analysis. The remaining cycles were used for data collection. Mice were habituated to the procedure for at least 3 days before blood pressure measurements were recorded.

### Echocardiography.

Mice were anesthetized with 1.5% isoflurane (1.5% oxygen) and imaged in the supine position using a Vevo 3100 Imaging System with a 35-MHz linear probe (Visualsonics). The core temperature was maintained at 37°C. Heart rates were kept consistent between the groups (400–450 bpm) (50). The heart was imaged in a 2D, short axis, and 4-chamber view by placing the pulsed wave Doppler at the septal corner of the mitral annulus. Mitral inflow velocity (E) and longitudinal tissue velocity of the mitral anterior annulus (e′) were measured through Vevo 2100 1.5.0 Image Software (51).

### Statistics.

Statistical analyses were performed using Prism software v8.4.1 (GraphPad). The Pearson-D’Agostino normality test was used to assess data distribution normality. When comparing 2 groups, a 2-tailed Student’s *t* test with Welch’s correction (unequal variances) was used. When comparing data for 2 or 3 interacting variables, 2-way and 3-way ANOVA tests were used, respectively. A *P* value of less than 0.05 was considered significant. When the number of data points was less than 10, data are presented as single values (dot plots, histograms). Analyses pooling data points over time are presented as line plots connecting means and SEM.

### Study approval.

Mice were housed in a pathogen-free facility in accordance with the American Veterinary Medical Association (AVMA) and under protocols fully approved by the Institutional Animal Care and Use Committee (IACUC) at CCHMC (nos. 2022-0020 and 2023-0002).

### Data availability.

RNA-seq and ChIP-seq datasets reported here are available in the NCBI GEO as GSE252826, GSE252857, GSE252856, GSE254114. Individual data for all charts presented here are available in the Supporting Data Values file.

## Author contributions

HBD, ADP, FEAS, HL, KMF, KM, KP, OA, and CW curated and analyzed data and conducted experiments. MKJ and SMH provided the *Klf15*-flox and *Klf15*^3xFLAG/wt^ mice, respectively. MQ conceptualized the study, analyzed data, acquired funding, and supervised the study. The order of co–first authors was assigned based on overall weight of contribution considering initial and revised submissions.

## Supplementary Material

Supplemental data

Unedited blot and gel images

Supporting data values

## Version 1 10/08/2024

In-Press Preview

## Version 2 11/22/2024

Electronic publication
