# Supplementary material for: Glucocorticoid chronopharmacology promotes glucose metabolism in heart through a cardiomyocyte-autonomous transactivation program
Source: JCI Insight. 2024 Nov 22;9(22):e182599. doi: 10.1172/jci.insight.182599 (PMC11601906; doi:10.1172/jci.insight.182599)
Supplement: Supplemental data [file jciinsight-9-182599-s063.pdf]

## Supplementary Figures

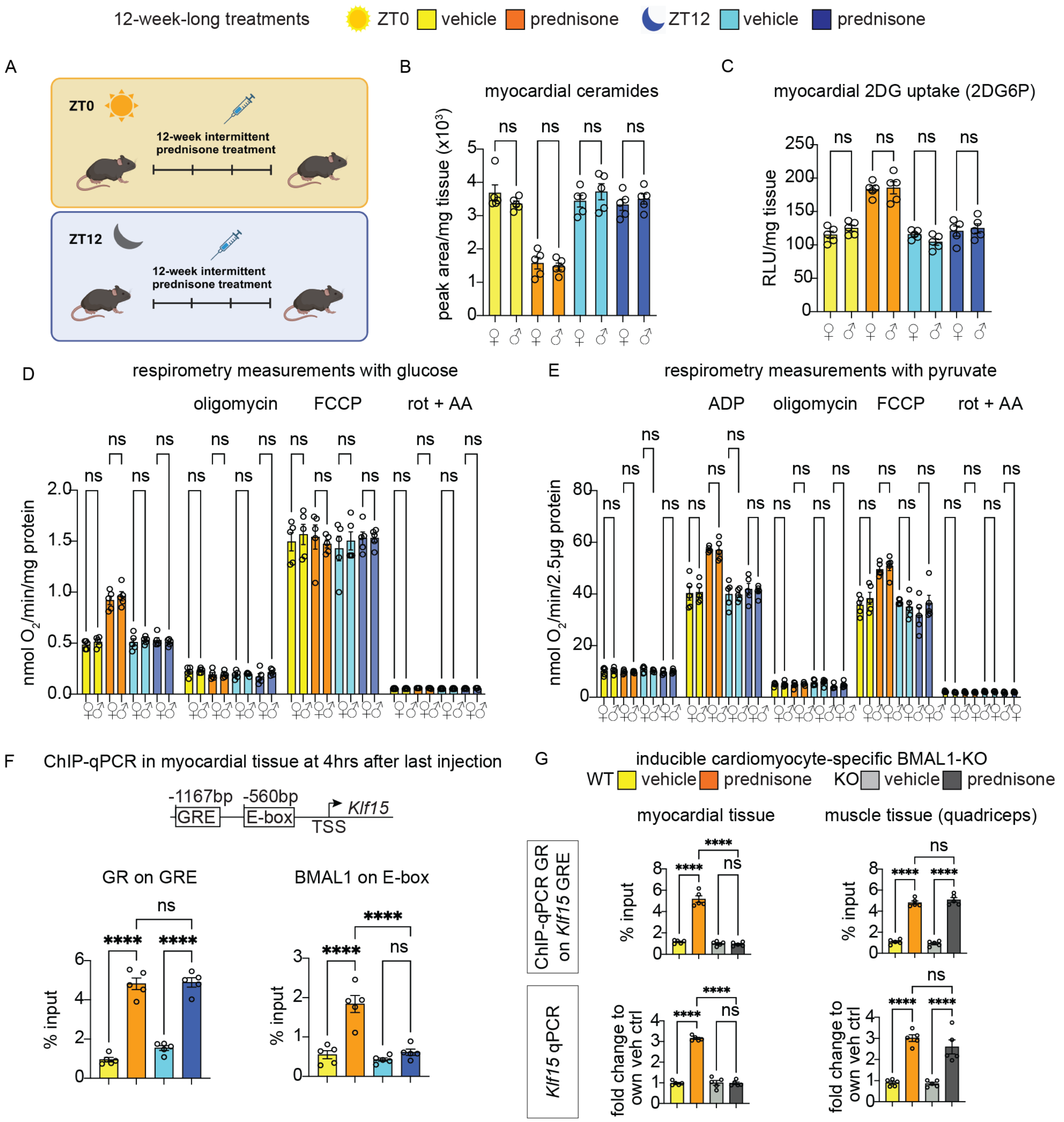


**Supplementary Figure 1. Additional analyses related to Figure 1. (A)** Graphical experimental design for wild-type mice. **(B)** Ceramide levels were decreased upon treatment with prednisone at ZT0, but not at ZT12. This effect was sex-independent. **(C)** Increased insulin-dependent 2DG uptake in heart showed no sexual dimorphism. **(D)** Although the cardiomyocytes showed an increase in basal glucose-fueled respiration ex vivo, no differences between the two sexes were observed. **(E)** ZT0 treatment increased ADP-stimulated respiration and respiratory control ratio with pyruvate. These effects were sex-independent. **(F)** While GR occupancy of *Klf15* promoter was increased by treatment independent from time-of-intake, ZT0 but not ZT12 treatment increased BMAL1 recruitment. **(G)** Ablation of BMAL1 in adult cardiomyocytes in vivo blocked the ZT0 prednisone-driven effects on GR occupancy of *Klf15* promoter and *Klf15* mRNA upregulation in heart (BMAL1 ablation) but not in muscle (no BMAL1 ablation). Shown are mean±S.E.M, histograms show also individual mouse values. n=(5♀+5♂)/group B-E, 5♂/group in F-G; 2w ANOVA+ Sidak; ns= non-significant. *, P<0.05; **, P<0.01; ***, P<0.001; ****, P<0.0001.

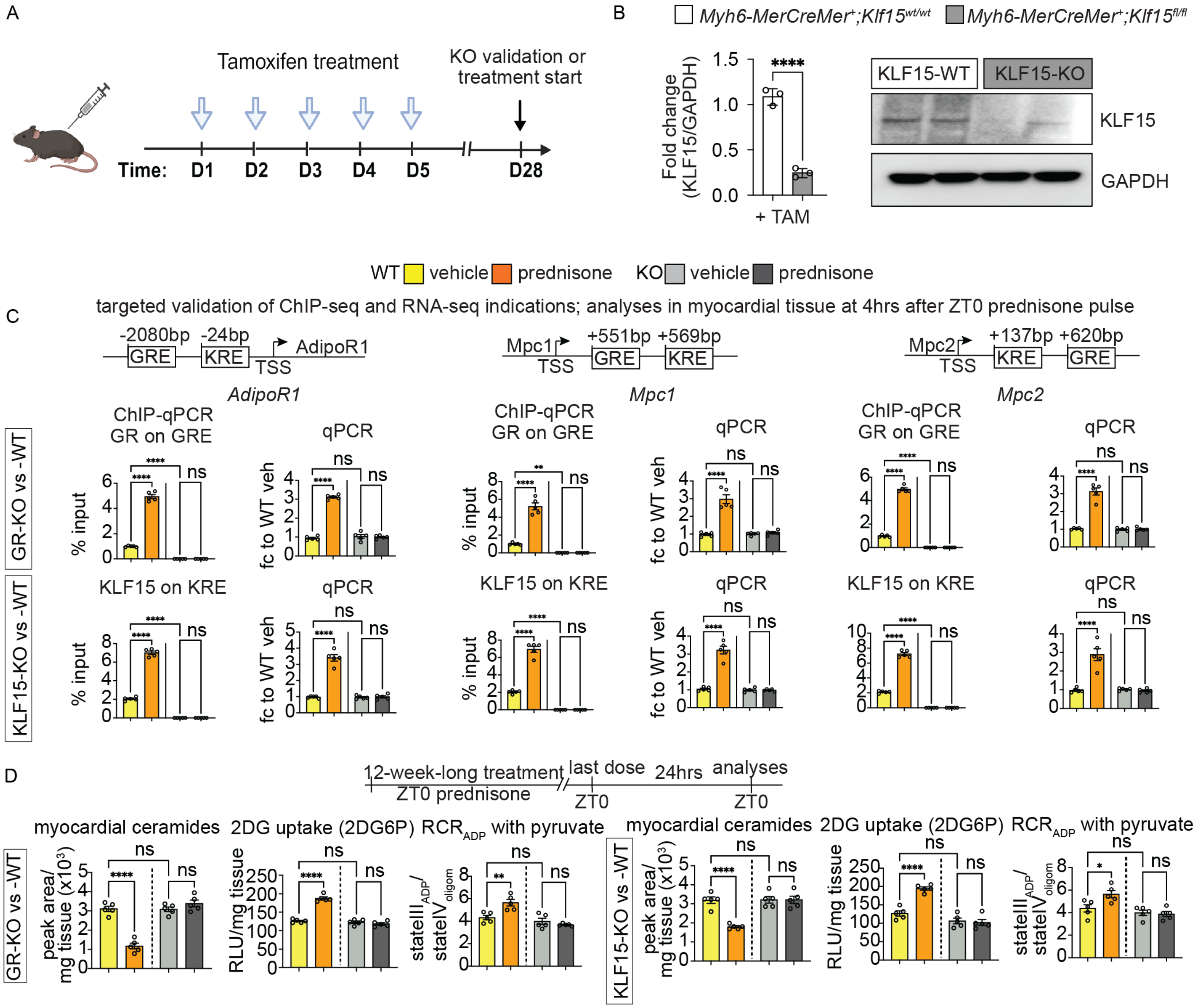


**Supplementary Figure 2. Tamoxifen regimen, KLF15-KO validation and light-phase effects of chronic treatments in GR-KO and KLF15-KO hearts. (A)** Schematic representing the generation and washout of tamoxifen-mediated inducible knockout mice for GR or KLF15. **(B)** Validation of KLF15 ablation in heart through WB. **(C)** Targeted validation through ChIP-qPCRs and qPCRs of ChIP-seq and RNA-seq indications of GR and KLF15 transactivation of *AdipoR1*, *Mpc1* and *Mpc2* in heart after ZT0 prednisone. **(D)** Related to Figure 3B-D. GR and KLF15 were both required for treatment effects on myocardial ceramides, glucose uptake and pyruvate oxidation at 24hrs from last ZT0 prednisone dose, i.e. in the rest phase. Shown are mean±S.E.M, histograms show also individual mouse values. B, n=3/group, Welch's t-test; C, n=5♂/group, 2w ANOVA + Sidak; *, P<0.05; **, P<0.01; ***, P<0.001; ****, P<0.0001.

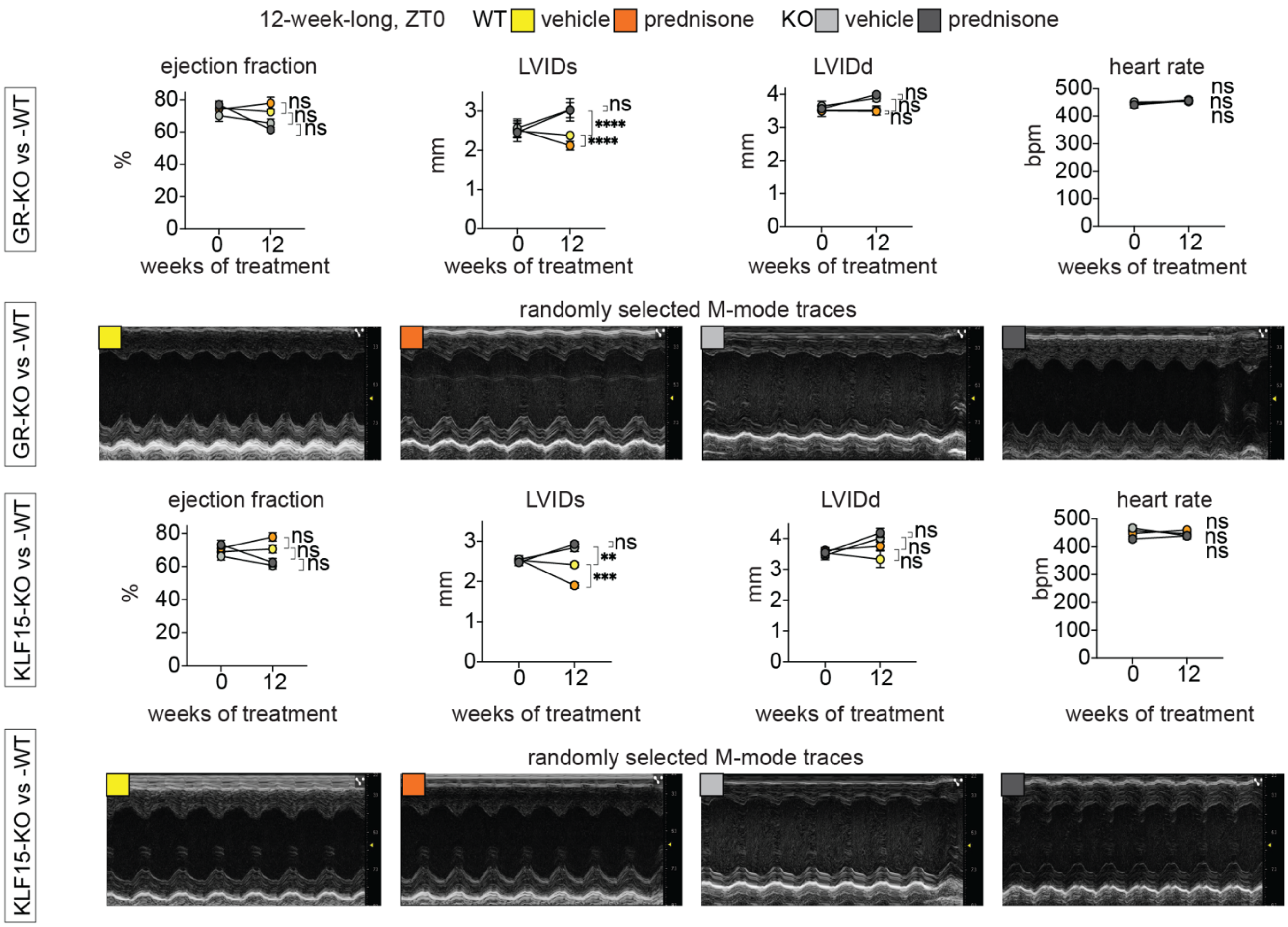


**Supplementary Figure 3. Additional echocardiographic measurements related to Figure 4.** In line with the trends in fractional shortening and stroke volume (Figure 4), inducible ablation of either GR or KLF15 in cardiomyocytes enlarged the systolic left ventricle diameter (LVIDs) and induced non-significant declines in ejection fraction and gains in diastolic left ventricle diameter (LVIDd). In WT hearts, treatment induced a small significant reduction of LVIDs, a non-significant trend in increased ejection fraction and no appreciable changes in LVIDd. In KO hearts, treatment had no sizable effects. Heart rate during the echocardiographic measurements, i.e. under anesthesia, was not changed by either KO or treatment. Shown are mean±S.E.M, histograms show also individual mouse values. N=6♂/group; curves, 3w ANOVA + Sidak; histograms, 2w ANOVA + Sidak; *, $P<0.05$; **, $P<0.01$; ***, $P<0.001$; ****, $P<0.0001$.

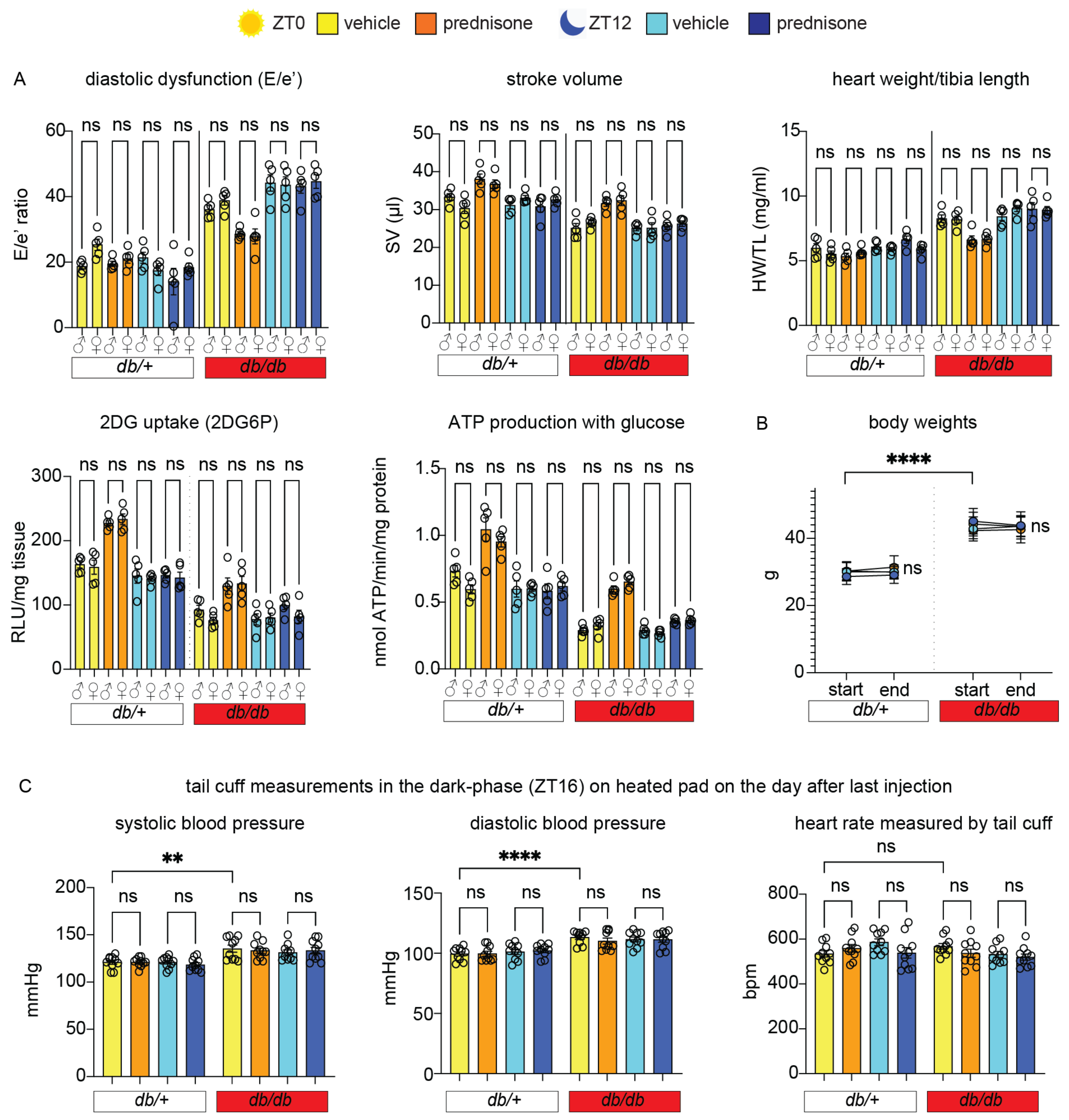


**Supplementary Figure 4. Additional measurements of cardiac function and blood pressure related to Figure 5. (A)** Sex-disaggregated measurements for male and female mice (*db/+*) and (*db/db*) for E/e'**,** stroke volume**,** heart weight/tibia length, 2DG uptake and ATP-linked respiration in cardiomyocytes showed no sizable sexual dimorphism in the treatment effects, either with ZT0 or ZT12 prednisone**. (B)** Treatments had no sizable effects on body weight, which was significantly increased by genotype. **(C)** Treatments had no sizable effects on blood pressure or heart rate during blood pressure measurements beyond the expected *db/db*-related mild hypertension in both systole and diastole. Shown are mean±S.E.M, histograms show also individual mouse values. n=(5♀+5♂)/group; 3w ANOVA + Sidak; *, P<0.05; **, P<0.01; ***, P<0.001; ****, P<0.0001.

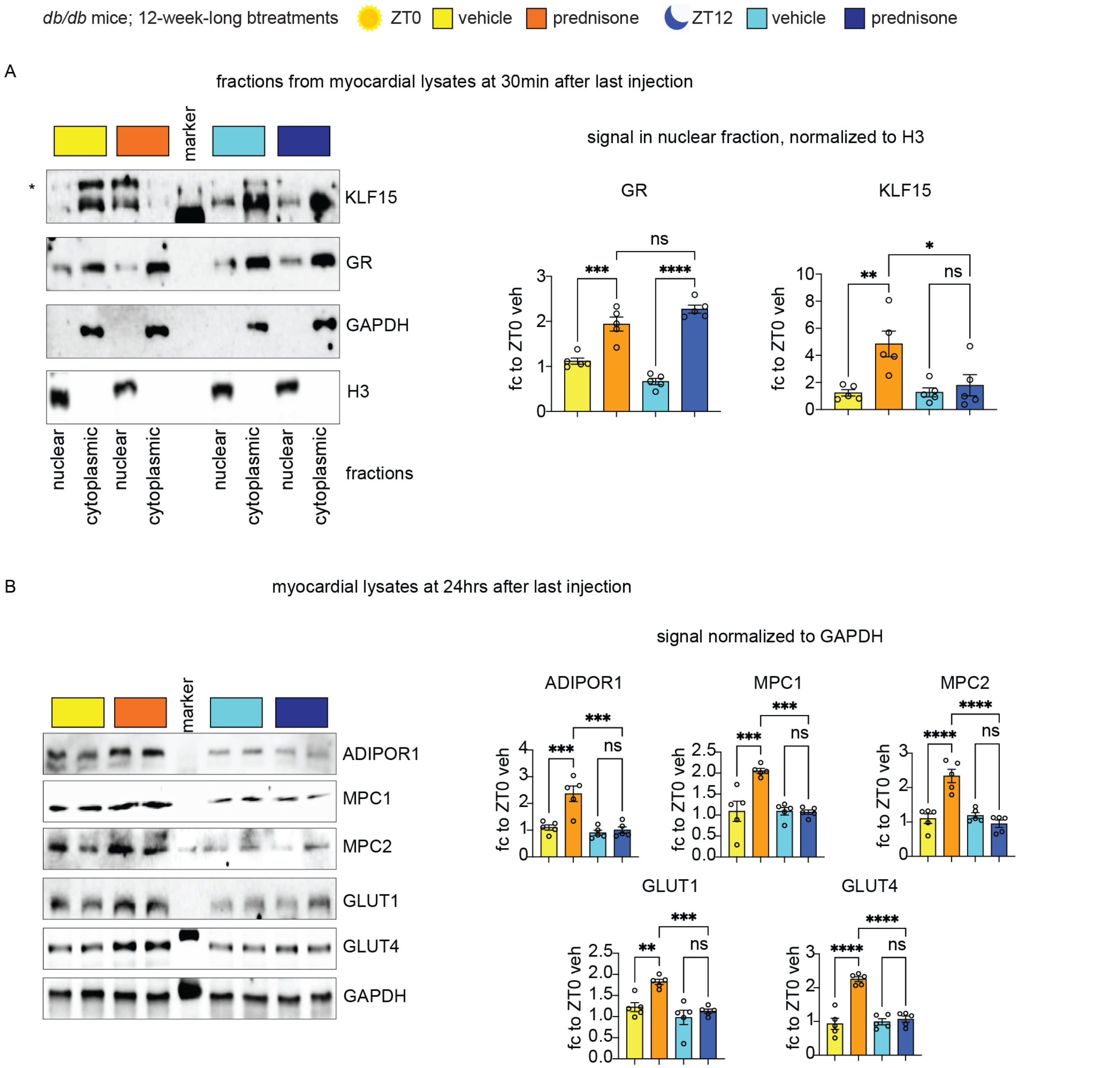


**Supplementary Figure 5. Additional protein analyses in *db/db* hearts related to Figure 5. (A)** WB in nuclear (H3⁺) and cytoplasmic (GAPDH⁺) fractions showed that time-of-intake changed treatment-driven nuclear translocation of KLF15 but not GR in *db/db* hearts. Asterisk denotes unspecific bands. **(B)** ZT0 intermittent prednisone – but not ZT12 – increased the myocardial protein levels of ADIPOR1, MPC1, MPC2, GLUT1 and GLUT4 in *db/db* mice compared to vehicle. Shown are mean±S.E.M, histograms show also individual mouse values. n=5♂/group, 2w ANOVA + Sidak; *, P<0.05; **, P<0.01; ***, P<0.001; ****, P<0.0001.
