# Supplementary material for: Glucocorticoid chronopharmacology promotes glucose metabolism in heart through a cardiomyocyte-autonomous transactivation program
Source: JCI Insight. 2024 Nov 22;9(22):e182599. doi: 10.1172/jci.insight.182599 (PMC11601906; doi:10.1172/jci.insight.182599)

Suppl. Figure 2B

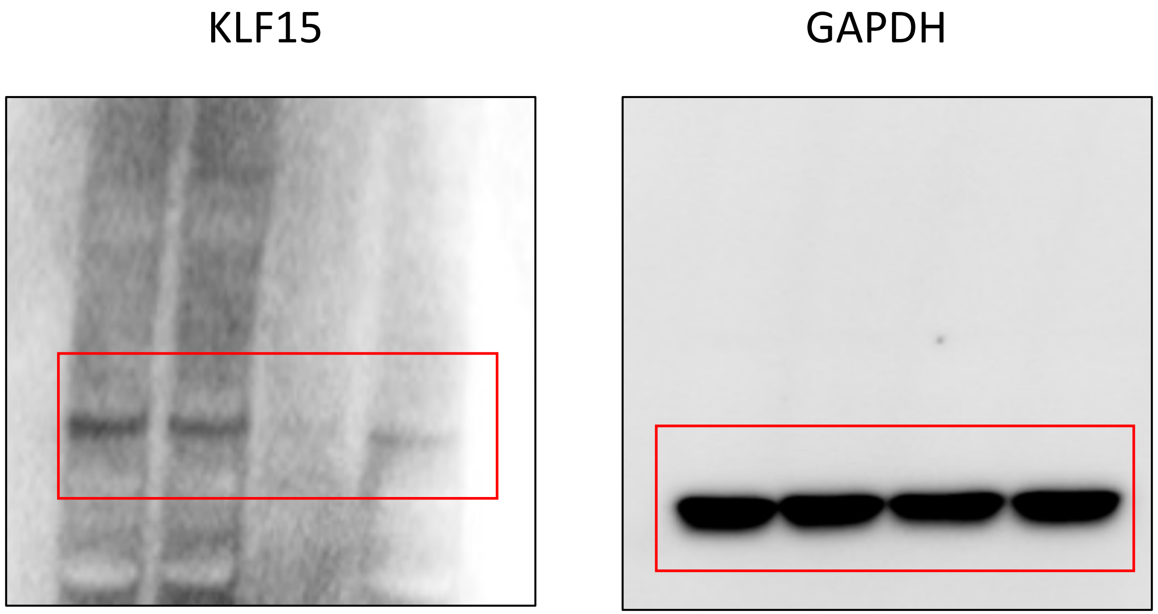

Figure 1C

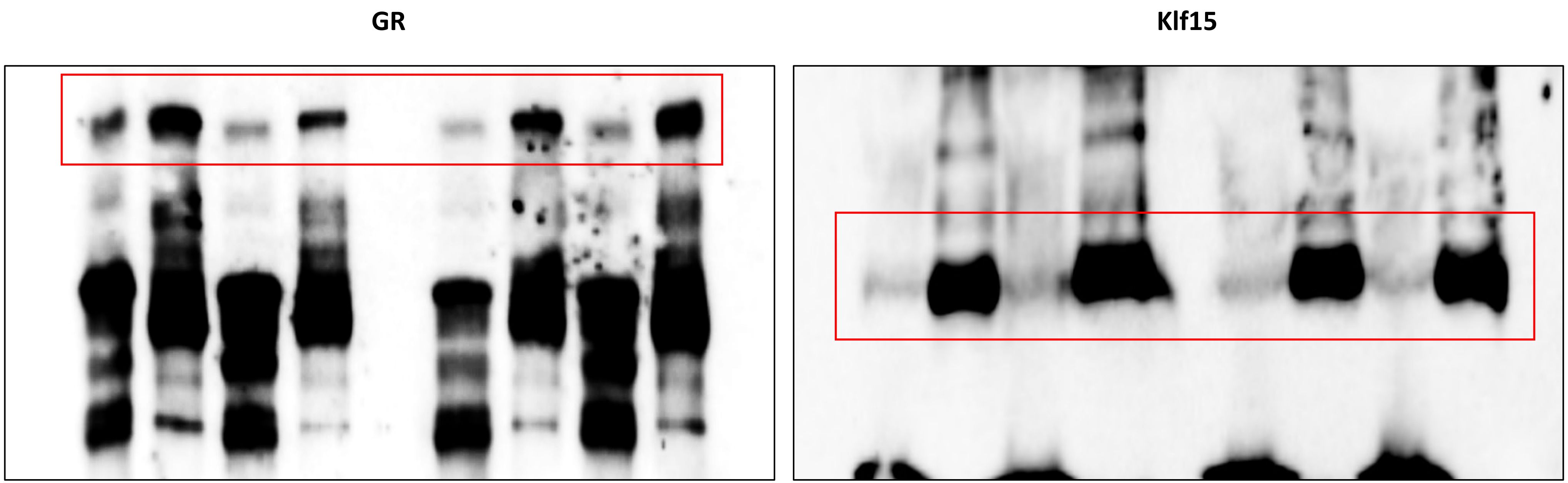

Suppl. Figure 5A

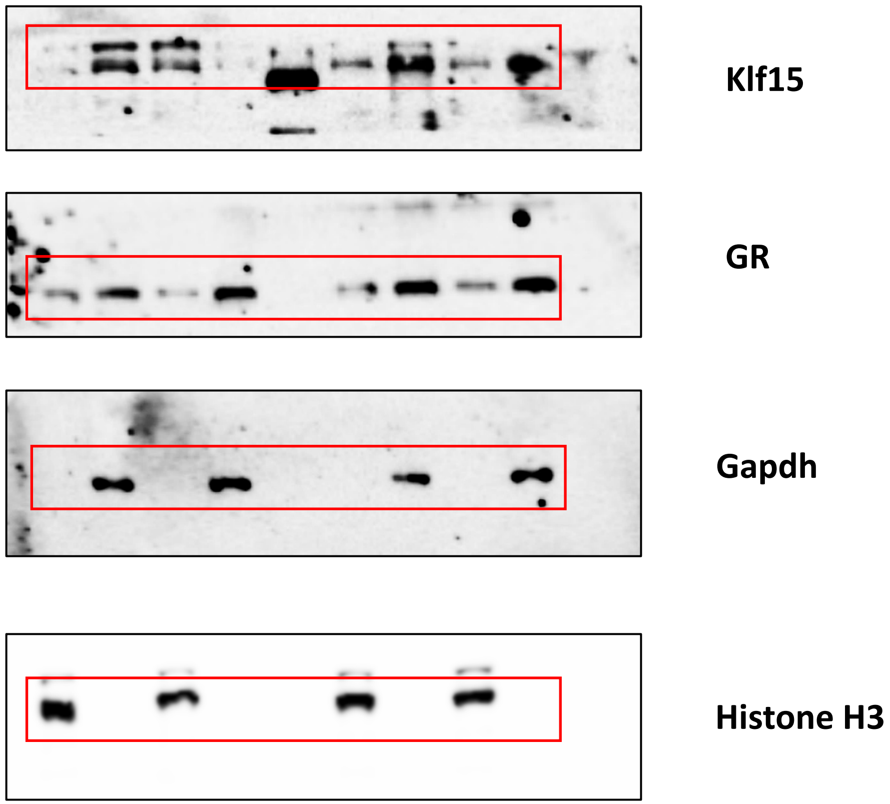

Suppl. Figure 5B

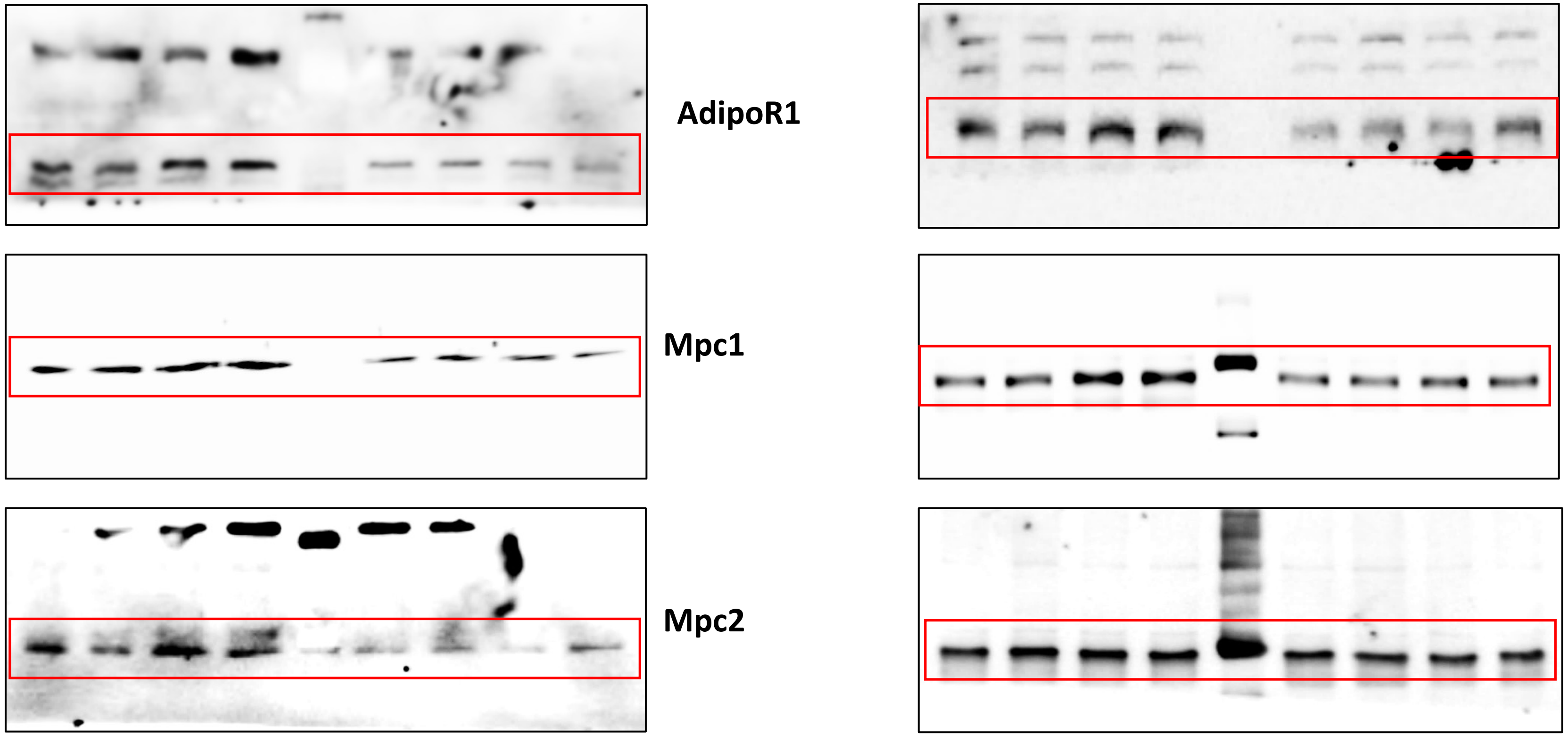

Figure 6A

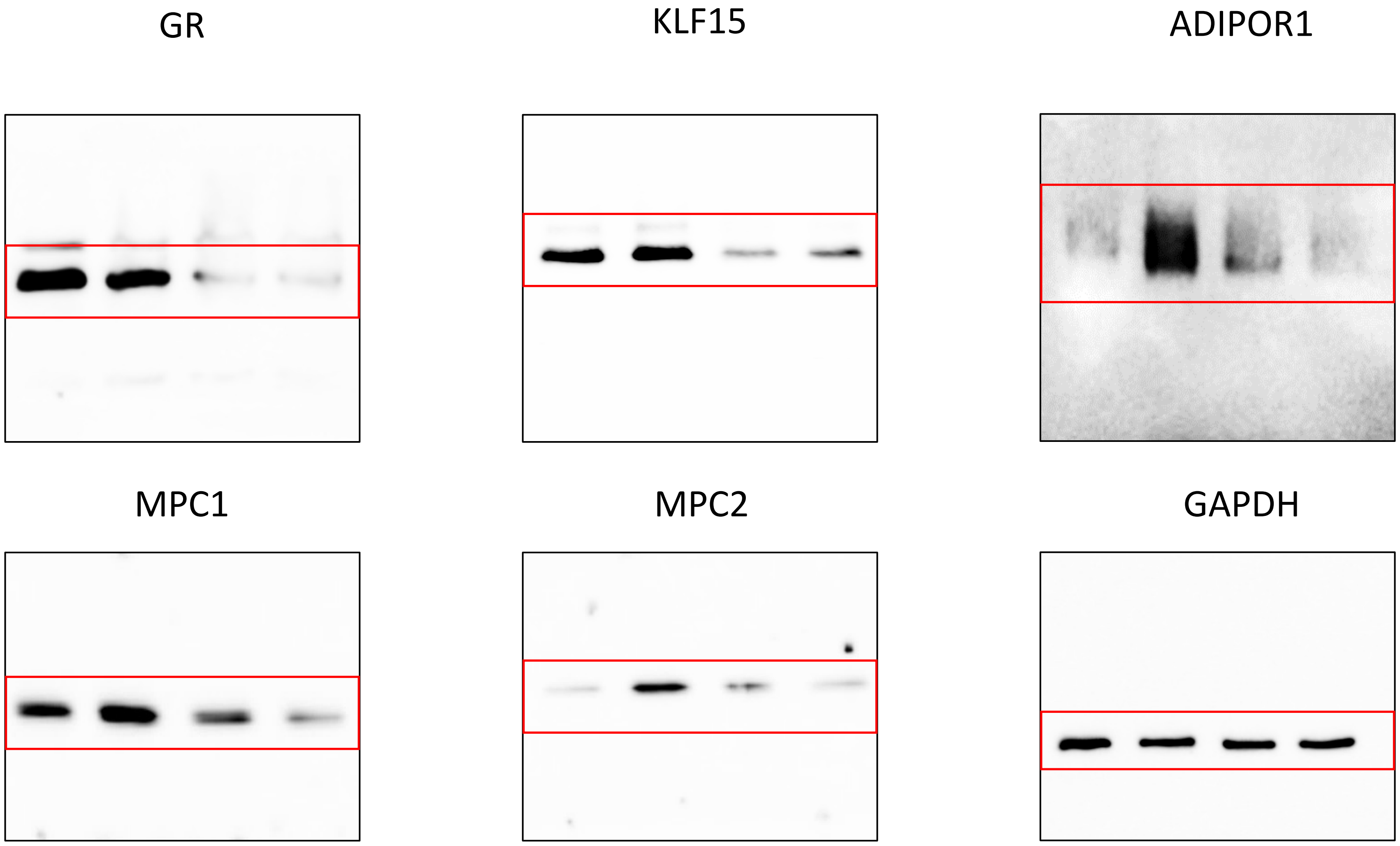

Supplement: Unedited blot and gel images [file jciinsight-9-182599-s064.pdf]
